# Enhanced Antimicrobial Efficacy of Sulfones and Sulfonamides via Cage-Like Silsesquioxane Incorporation

**DOI:** 10.1021/acs.inorgchem.4c05156

**Published:** 2025-03-25

**Authors:** Kamila Fuchs, Tomasz Janek, Mateusz Karpl, Anna Władyczyn, Jolanta Ejfler, Łukasz John

**Affiliations:** † Faculty of Chemistry, University of Wrocław, 14 F. Joliot-Curie, 50-383 Wrocław, Poland; ‡ Department of Biotechnology and Food Microbiology, Wrocław University of Environmental and Life Sciences, 37 Chełmońskiego, 51-630 Wrocław, Poland

## Abstract

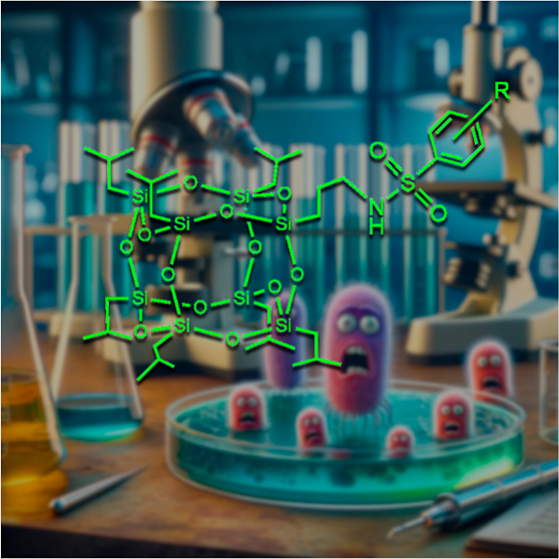

This work introduces a novel class of hybrid antimicrobial agents by integrating sulfone and sulfonamide functionalities with polyhedral oligomeric silsesquioxanes (POSSs). By employing efficient synthetic protocols, we have successfully prepared both sulfone (ethylvinylsulfone-POSS and phenylethylsulfone-POSS) and sulfonamide (benzenesulfonamide-POSS, *p*-toluenesulfonamide-POSS, 3-fluorobenzenesulfonamide-POSS, and 2-naphthalenesulfonamide-POSS) derivatives with high yields (73–90%). All derivatives were examined using Fourier transform infrared spectroscopy, multinuclear (^1^H, ^13^C, ^19^F, and ^29^Si) NMR spectroscopy, MALDI-ToF MS spectrometry, and elemental analysis. Additionally, the crystal structure of the *p*-toluenesulfonamide-POSS hybrid was revealed. The unique cage-like POSS structure not only imparts enhanced thermal and chemical stability, a common feature of silsesquioxane-based hybrids, but also boosts the lipophilic character of these compounds, thereby facilitating their interaction with microbial membranes. This interaction, likely resulting in membrane disruption and cell lysis, translates into potent antimicrobial activity (against *Escherichia coli*, *Pseudomonas aeruginosa*, *Enterococcus hirae*, *Staphylococcus aureus*, and *Candida albicans*)—especially against Gram-positive bacteria—at remarkably low minimum inhibitory concentrations in the range from 125 to 3000 μM. In turn, *E. hirae* and *S. aureus* were more susceptible compared to Gram-negative bacteria and *C. albicans*. The strategic incorporation of POSSs into these sulfur-based moieties represents a significant breakthrough, opening new avenues for the development of advanced antimicrobial coatings and therapeutic agents in the fight against antibiotic resistance.

## Abstract 2

This work presents hybrid antimicrobial agents synthesized by integrating cage-like silsesquioxanes (POSSs) with sulfone and sulfonamide functionalities. The unique POSS framework enhances stability and lipophilicity, promoting effective interactions with microbial membranes and resulting in potent activity, especially against Gram-positive bacteria. These findings open exciting avenues for developing antimicrobial coatings and therapies in the era of rising antibiotic resistance.

## Introduction

Sulfones and sulfonamides are valuable in chemistry and pharmaceuticals due to their notable biological activity, stability, and synthetic accessibility. Some sulfonamides serve as antibiotics, while sulfones treat diseases like malaria and leprosy.^1^ Their stability under various conditions makes them suitable for diverse chemical reactions and pharmaceutical formulations.^2,3^ They are often synthesized from readily available materials, enhancing cost-effectiveness.^4^ Many sulfonamide drugs exhibit good oral bioavailability and metabolic stability, improving their pharmaceutical efficacy.^5^ Their ability to selectively target enzymes or receptors enables potent therapeutic agents.^6,7^ Extensive research has established structure–activity relationship data, aiding in designing bioactive compounds.^8^ Beyond pharmaceuticals, sulfones and sulfonamides are used in agrochemicals and materials science due to their versatile properties.^9,10^ This inherent versatility facilitates the generation of a diverse array of chemical structures and sometimes unconventional synthetic approaches,^11−14^ a pivotal aspect inter alia in drug discovery and development.^15−18^ However, conventional sulfone and sulfonamide compounds sometimes face limitations in addressing emerging challenges, such as antibiotic resistance and the need for multifunctional materials with improved performance.

Recent advances in hybrid materials have provided innovative strategies to overcome these limitations by integrating bioactive moieties with silicon-based frameworks.^19−22^ Among silicon-containing hybrid materials, polyhedral oligomeric silsesquioxanes (POSSs) are attracting significant attention due to their unique cage-like structure, which provides enhanced thermal and chemical stability, increased lipophilicity, and exceptional versatility in functionalization. Introducing POSS, for instance, into polymers allows for the creation of materials with highly specialized properties. As a result, these systems find applications across a wide range of fields from modern dressings and implants to smart drug delivery systems. Here, precise control of the composition and morphology of the material is crucial to achieve the desired biological and functional effects affecting biocompatibility, antibacterial properties, promoting cell adhesion, reducing inflammation, antiviral and antifungal properties, and modifying drug release.^23,24^ Recent findings from our research group have demonstrated that hybrid materials based on these particular organosilicons lead to a wide range of applications in materials engineering,^25−28^ tissue engineering,^29^ drug delivery systems,^30^ and coordination chemistry.^31^

Yet, the full potential of POSS-based sulfones and sulfonamides as advanced antimicrobial agents remains underexplored. In this context, Ziarani et al. reported the modification of octa-substituted POSSs with multifunctional sulfonamide groups via a click condensation approach.^32^ These POSS-like derivatives were synthesized through a copper-catalyzed Huisgen 1,3-dipolar cycloaddition reaction, involving the direct propargylation of sulfonamides, subsequent azide exchange of POSS-(Cl)_8_ with sodium azide, and a final click reaction facilitated by CuI in DMF. While the resulting structures exhibited antimicrobial activity, their effectiveness was diminished due to increased particle mass and reduced solubility.

This study introduces a scalable and efficient synthetic approach to developing antimicrobial agents by integrating sulfone and sulfonamide functionalities with monosubstituted POSS moieties. Direct incorporation into the POSS framework streamlines synthesis, while preserving and enhancing the bioactivity of sulfur-based groups. The rigid POSS core improves material stability and promotes microbial membrane interactions, where its lipophilicity facilitates penetration, leading to membrane disruption and cell lysis, particularly in Gram-positive bacteria. The observed antimicrobial activity highlights the potential of POSS-based hybrids for advanced coatings and therapeutics. By strategically embedding sulfone and sulfonamide groups within the POSS structure, our approach overcomes the limitations of conventional sulfur-containing compounds, yielding hybrid materials with significantly enhanced antimicrobial properties. The high-yield synthesis, performed under mild conditions, ensures scalability and sustainability, addressing the urgent demand for new antimicrobial solutions in response to rising antibiotic resistance. This innovative platform not only advances antimicrobial strategies but also opens new avenues for multifunctional materials in medicine, environmental safety, and beyond.

## Experimental Section

### Materials and Methods

All commercially available chemicals were used without further purification: 2,2-Dimethoxy-2-phenylacetophenone DMPA (Aldrich, 99%), divinylsulfone (Aldrich, ≥98%), phenylvinylsulfone (Aldrich, 99%), triethylamine (Chempur, 99%), *p*-toluenesulfonyl chloride (Aldrich, ≥99%), 3-fluorobenzenesulfonyl chloride (AmBeed, 99.92%), benzenesulfonyl chloride (Thermo Fisher Scientific, 98%), 2-naphthalenesulfonyl chloride (Thermo Fisher Scientific, 97%), 3-mercaptopropylheptaisobutyl-POSS (Hybrid Plastics Inc.), and 3-aminopropylheptaisobutyl-POSS (Hybrid Plastics Inc.). Solvents for synthesis: toluene and methylene chloride (HPLC, VWR) were dried and purified by using the solvent purification systems (Inert, PureSolv EN 1–7 Base). Solvents for the standard workup were used as received. The ^1^H and ^13^C NMR spectra were obtained at 300 K by using a BrukerAvance III 500 MHz spectrometer. The ^29^Si NMR spectra were obtained at 300 K using a BrukerAvance III 600 MHz spectrometer. The chemical shifts are given in ppm relative to the residual signals of the solvent (CDCl_3_, ^1^H: 7.26 ppm, ^13^C: 77.16 ppm). The ^19^F NMR spectra was obtained at 300 K using a Jeol JNM-ECZR 500 MHz spectrometer. HRMS spectra were recorded using JEOL JMS-S3000 SpiralTOF-plus Ultra-High Mass Resolution MALDI-ToF MS. Infrared (IR) spectra were recorded using a Bruker Vertex 70 spectrometer (measured samples were prepared by making a KBr pellet) and a Shimadzu IR Spirit-T spectrometer with a diamond ATR attachment. Elemental analyses were measured on an Elementar’s Vario EL Cube analyzer. Crystallographic data of **4** were obtained using an XtaLAB Synergy R, DW system, HyPix-Arc 150 diffractometer by using graphite-monochromatized Cu Kα radiation (λ = 1.54184 Å) at 100 K. Frame integration, data reduction, and absorption correction were performed using the CrysAlisPro^33^ program package. Using Olex^2^ software,^34^ the structure was solved with the SHELXS^35^ structure solution program using Direct Methods and refined with the SHELXL^36^ refinement package using least-squares minimization. The positions of the hydrogen atoms were idealized using the HFIX command. Details of the crystal parameters, data collection, and refinement for structure of **4** are listed in Table 1. Crystallographic data have been deposited with the Cambridge Crystallographic Data Centre (CCDC) as supplementary publication CCDC 2355504. Copies of the data can be obtained free of charge by application to CCDC, 12 Union Road, Cambridge CB2 1EZ, UK [fax: (Internet.) + 44 1223/336–033; e-mail: deposit@ccdc.cam.ac.uk].

**Table 1:** Selected Experimental Details for **4**

	4
deposition number	2355504
empirical formula	C_38_H_77_NO_14_SSi_8_
formula weight	1028.78
crystal system	Triclinic
space group	*P*1̅
temperature/K	100(2)
*a*, *b*, *c*/Å	9.967(10), 10.950(2), 24.232(3)
α, β, γ/°	91.28(10), 91.70(10), 91.21(10)
volume/Å^3^	2642.2(6)
*Z*	2
radiation	Cu Kα
μ/mm^–1^	1.24
crystal size/mm^3^	0.32 × 0.24 × 0.16
*R*_int_	0.061

### Syntheses

All of the reactions and operations that required an inert atmosphere were performed by using a Schlenk apparatus and vacuum line techniques. The following procedures are described for laboratory scale, but these syntheses have also been tested on a larger scale, where the main product was obtained in approximately 5 g. The solubility of the resulting **1–6** in commonly used solvents is described in Table S2.

Note: No uncommon hazards are noted.

### Synthesis of Ethylvinylsulfone-POSS (**1**)

In a round-bottom flask equipped with a magnetic stirrer, 0.2 mmol (180 mg) of 3-mercaptopropylheptaisobutyl-POSS (precursor **A**) and 0.02 mmol (5 mg) of catalyst (DMPA) were dissolved in toluene and left on the stirrer for 15 min. Then, 0.2 mmol (24 mg, 20 μL) of divinylsulfone was slowly added dropwise. The flask was placed under a UV lamp (365 nm) for 24 h. After this time, the mixture was evaporated using a rotary evaporator. The product was precipitated by washing it three times with cold methanol. The workup gave a white precipitate with 90% yield. ^1^H NMR (500 MHz, CDCl_3_, 300 K): δ 6.65 (m, 1H), 6.45 (d, *J*_H,H_ = 16.6 Hz, 1H), 6.18 (d, *J*_H,H_ = 9.9 Hz, 1H), 3.19 (m, 2H), 2.82 (m, 2H), 2.54 (m, 2H), 1.84 (m, 7H), 1.67 (m, 2H), 0.94 (dd, *J*_H,H_ = 6.5, 2.0 Hz, 42H), 0.68 (m, 2H), 0.58 (dd, *J*_H,H_ = 6.8, 3.0 Hz, 14H). ^13^C NMR (126 MHz, CDCl_3_, 300 K): δ 136.15 (s, 1C), 131.04 (s, 1C), 54.62 (s, 1C), 35.26 (s, 1C), 25.69 (s, 14C), 24.06 (s, 1C), 23.85 (s, 7C), 22.96 (s, 1C), 22.50 (s, 7C), 11.58 (s, 1C). ^29^Si NMR (99 MHz, CDCl_3_, 300 K): δ −67.63, −67.86, −68.24. MALDI-MS: *m*/*z*: 1031.28 {calcd [M + Na]^+^ 1031.28}. IR (cm^–1^): ν_C–H_ = 2955, (s), ν_C–H_ = 2928 (w), ν_C=C_ = 1634 (m), δ_S–O_ = 1327 (m), ν_Si–C_ = 1231 (s), ν_Si–O__–__Si_ = 1110 (vs), ν_S–C_ = 743. Elemental analyses calcd (%); found, for C_35_H_76_O_14_S_2_Si_8_: C, 41.63 (41.43); H, 7.59 (7.53); S, 6.35 (6.23).

### Synthesis of Phenylethylsulfone-POSS (**2**)

Synthesis was carried out analogously to that for **1**, but instead of divinylsulfone, 0.2 mmol (34 mg) of phenylvinylsulfone was added. The workup gave a white precipitate with 73% yield. ^1^H NMR (500 MHz, CDCl_3_, 300 K): δ 7.90 (m, 2H), 7.66 (m, 1H), 7.56 (m, 2H), 3.31 (m, 2H), 2.75 (m, 2H), 2.48 (t, *J*_H,H_ = 7.3 Hz, 2H), 1.83 (m, 7H), 1.62 (m, 2H), 0.93 (d, *J*_H,H_ = 6.6 Hz, 42H), 0.65 (m, 2H), 0.58 (d, *J*_H,H_ = 7.0 Hz, 14H). ^13^C NMR (126 MHz, CDCl_3_, 300 K): δ 138.94 (s, 1C), 133.82 (s, 1C), 129.39 (s, 2C), 127.99 (s, 2C), 56.52 (s, 1C), 35.20 (s, 1C), 25.68 (s, 14C), 24.26 (s, 1C), 23.84 (d, 7C), 22.89 (s, 1C), 22.45 (d, 7C), 11.51 (s, 1C). ^29^Si NMR (99 MHz, CDCl_3_, 300 K): δ −67.65, −67.88, −68.93. MALDI-MS: *m*/*z*: 1081.29 {calcd [M + Na]^+^ 1081.28}. IR (cm^–1^): ν_C–H_ = 2950 (w), ν_C–H_ = 2927 (w), δ_S–O_ = 1368 (m), ν_Si–C_ = 1230 (m), ν_Si–O__–__Si_ = 1083 (vs), δ_Ar_ = 839 (m), ν_S–C_ = 736 (m), δ_O–Si__–__C_ = 471 (s). Elemental analyses calcd (%); found, for C_39_H_78_O_14_S_2_Si_8_: C, 44.20 (44.03); H, 7.42 (7.31); S, 6.05 (5.89).

### Synthesis of Benzenesulfonamide-POSS (**3**)

In a round-bottom flask equipped with a magnetic stirrer, 0.2 mmol (35 mg, 26 μL) of benzenesulfonyl chloride and 0.2 mmol (20 mg, 28 μL) of triethylamine were dissolved in DCM and left on the stirrer for 15 min, and then, 0.15 mmol (131 mg) of 3-aminopropylheptaisobutyl-POSS (precursor **B**) was slowly added. The mixture was stirred under an inert atmosphere for 24 h. After this time, the solution was concentrated using an evaporator. The product was precipitated by washing several times with cold methanol. The workup gave a white precipitate with 76% yield. ^1^H NMR (500 MHz, CDCl_3_, 300 K): δ 7.84 (m, 2H), 7.55 (m, 1H), 7.49 (m, 2H), 4.31 (s, 1H), 2.95 (m, 2H), 1.83 (m, 7H), 1.55 (m, 2H), 0.94 (dd, *J*_H,H_ = 6.6, 4.8 Hz, 42H), 0.57 (m, 16H). ^13^C NMR (126 MHz, CDCl_3_, 300 K): δ 140.17 (s, 1C), 132.53 (s, 1C), 129.06 (s, 2C), 126.99 (s, 2C), 45.53 (s, 1C), 25.67 (s, 14C), 23.85 (s, 7C), 23.33 (s, 1C), 22.44 (s, 7C), 9.17 (s, 1C). ^29^Si NMR (99 MHz, CDCl_3_, 300 K): δ −67.60, −67.87, −68.21. MALDI-MS: *m*/*z*: 1036.28 {calc. [M + Na]^+^ 1036.30}. IR (cm^–1^): ν_N–H_ = 3266 (w), ν_C–H_ = 2955 (m), ν_C–H_ = 2869 (w), δ_S–O_ = 1328 (m), ν_Si–C_ = 1230 (s), ν_Si–O__–__Si_ = 1086 (vs), δ_Ar_ = 833 (w), ν_S–C_ = 741(s), δ_O–Si__–__C_ = 465 (s). Elemental analyses calcd (%); found, for C_37_H_75_NO_14_S_2_Si_8_: C, 43.80 (43.91); H, 7.45 (7.53); N, 1.38 (1.27); S, 3.16 (3.03).

### Synthesis of *p*-Toluenesulfonamide-POSS (**4**)

Synthesis was carried out analogously to that for **3**, but instead of benzenesulfonyl chloride, 0.2 mmol (38 mg) of *p*-toluenesulfonyl chloride was added. The workup gave a white precipitate with 85% yield. ^1^H NMR (500 MHz, CDCl_3_, 300 K): δ 7.71 (m, 2H), 7.27 (m, 2H), 4.33 (t, *J*_H,H_ = 6.1 Hz, 1H), 2.92 (q, *J*_H,H_ = 6.7 Hz, 2H), 2.38 (s, 3H), 1.82 (m, 7H), 1.52 (m, 2H), 0.92 (m, 44H) 0.57 (m, 14H). ^13^C NMR (126 MHz, CDCl_3_, 300 K): δ 143.24 (s,1C), 137.24 (s, 1C), 129.65 (s, 1C), 127.07 (s, 1C), 45.51 (s, 1C), 30.90 (s, 1C), 25.68 (s, 14C), 23.87 (s, 7C), 23.30 (s, 1C), 22.48 (s, 7C), 21.49 (s, 1C). ^29^Si NMR (99 MHz, CDCl_3_, 300 K): δ −67.60, −67.80, −68.17. MALDI-MS: *m*/*z*: 1050.31 {calcd [M + Na]^+^ 1050.32}; 1066.28 {calc. [M + K]^+^ 1066.42}. Elemental analyses calcd (%); found, for C_38_H_77_NO_14_SSi_8_: C, 44.37 (44.23); H, 7.54 (7.51); N, 1.36 (1.42) S, 3.12 (3.19). IR (cm^–1^): ν_N–H_ = 3260 (w), ν_C–H_ = 2955 (m), ν_C–H_ = 2869 (w), δ_S–O_ = 1328 (m), ν_Si–C_ = 1230 (m), ν_Si–O__–__Si_ = 1086 (vs), δ_Ar_ = 838 (w), ν_S–C_ = 741, and δ_O–Si__–__C_ = 473 (s).

### Synthesis of 3-Fluorobenzenesulfonamide-POSS (**5**)

Synthesis was carried out analogically as for **3**, but instead of benzenesulfonyl chloride, 0.2 mmol (40 mg, 27 μL) of 3-fluorobenzenesulfonyl chloride was added. The workup gave a white precipitate with 78% yield. ^1^H NMR (500 MHz, CDCl_3_, 300 K): δ 7.63 (d, *J*_H,H_ = 7.8 Hz, 1H), 7.55 (dt, *J*_H,H_ = 12.2, 8.1, 4.1 Hz, 1H), 7.48 (m, 1H), 7.26 (m, 1H), 4.39 (s, 1H) 2.96 (q, *J*_H,H_ = 6.8 Hz, 2H), 1.83 (m, 7H), 1.55 (m, 2H), 0.94 (m, 42H) 0.55 (m, 16H). ^13^C NMR (126 MHz, CDCl_3_, 300 K): δ 162.46 (d, *J*_C,C_ = 251.5 Hz, 1C), 142.40 (s, 1C), 130.89 (s, 1C), 122.70 (s, 1C), 119.64 (s, 1C), 114.51 (s, 1C), 45.57 (s, 1C), 25.64 (s, 14C), 23.87 (s, 7C), 23.37 (s, 1C), 22.47 (s, 7C), 9.21 (s, 1C). ^29^Si NMR (99 MHz, CDCl_3_, 300 K): δ −67.58, −67.86, −68.29. ^19^F NMR (470 MHz, CDCl_3_, 300 K): δ −109.52 (ddd, 1F, J_F–H(A)_ = 16.6 Hz; J_F–H(B)_ = 8.5 Hz; J_F–H(C)_ = 5.0 Hz). MALDI-MS: *m*/*z*: 1054.29 {calcd [M + Na]^+^ 1054.30}. Elemental analyses calcd (%); found, for C_37_H_74_FNO_14_SSi_8_: C, 43.03 (42.98); H, 7.22 (7.16); N, 1.36 (1.40); S, 3.10 (3.12). IR (cm^–1^): ν_N–H_ = 3266 (w), ν_C–H_ = 2955 (m), ν_C–H_ = 2869 (w), ν_S–O_ = 1334 (m), ν_Si–C_ = 1230 (s), ν_Si–O__–__Si_ = 1081 (vs), δ_Ar_ = 833 (s), δ_S–C_ = 741 (m), and δ_O–Si__–__C_ = 477 (s).

### Synthesis of 2-Naphthalenesulfonamid-POSS (**6**)

Synthesis was carried out analogically as for **3**, but instead of benzenesulfonyl chloride, 0.2 mmol (45 mg) of 2-naphthalenesulfonyl chloride was added. The workup gave a white precipitate with 82% yield. ^1^H NMR (500 MHz, CDCl_3_, 300 K): δ 8.41 (s, 1H), 7.95 (m, 2H), 7.88 (d, *J*_H,H_ = 7.9 Hz, 1H) 7.81 (m, 1H), 7.61 (m, 2H), 4.40 (m, 1H), 2.96 (q, *J*_H,H_ = 6.8 Hz, 2H), 1.82 (m, 7H), 1.55 (m, 2H), 0.92 (m, 42H) 0.54 (m, 16H). ^13^C NMR (126 MHz, CDCl_3_, 300 K): δ 136.96 (s, 1C), 134.79 (s, 1C), 132.20 (s, 1C), 129.33 (d, *J*_C,C_ = 27.7 Hz, 2C), 128.73 (s, 1C), 128.38 (s, 1C), 127.72 (d, *J*_C,C_ = 45.9 Hz, 2C), 122.32 (s, 1C), 45.58 (s, 1C), 25.67 (d, *J*_C,C_ = 5.6 Hz, 14C), 23.84 (d, *J*_C,C_ = 3.5 Hz, 7C), 23.36 (s, 1C) 22.47 (d, *J*_C,C_ = 10.6 Hz, 7C) 9.20 (s, 1C). ^29^Si NMR (99 MHz, CDCl_3_, 300 K): δ −67.60, −67.88, −68.22. MALDI-MS: *m*/*z*: 1086.31 {calcd [M + Na]^+^ 1086.32}. Elemental analyses calcd (%); found, for C_41_H_77_NO_14_SSi_8_: C, 46.25 (46.51); H, 7.29 (7.43); N, 1.32 (1.24); S, 3.01 (2.93). IR (cm^–1^): ν_N–H_ = 3258 (w), ν_C–H_ = 2955 (w), ν_C–H_ = 2875 (w), δ_S–O_ = 1328 (m), ν_Si–C_ = 1224 (w), ν_Si–O__–__Si_ = 1090 (vs), δ_Ar_ = 839 (w), ν_C–S_ = 741 (m), δ_O–Si__–__C_ = 465 (s).

### Biological Assessment

#### In Vitro Antimicrobial Activity

All strains were obtained from the American Type Culture Collection (ATCC, USA). The antimicrobial activity of the tested compounds were assayed using the following, Gram-negative bacteria: *Escherichia coli* ATCC 25922, *Pseudomonas aeruginosa* ATCC 15422, Gram-positive bacteria: *Enterococcus hirae* ATCC 10541, *Staphylococcus aureus* ATCC 25923, and yeast *Candida albicans* ATCC 10231. The strains were stored at –80 °C as a glycerol stock in the Department of Biotechnology and Food Microbiology, Wrocław University of Environmental and Life Sciences, Wrocław, Poland. The minimum inhibitory concentration (MIC) values of tested agents (at a concentration range of 50–3000 μM) were determined using the protocol recommended by the National Committee for Clinical Laboratory Standards.^37^ The microtiter plate wells were inoculated with 1 μL per well of a 24 h culture of microorganisms at a final cell density of 5 × 10^7^ colony forming units (CFU)/mL. The tested agents were dissolved in 100% DMSO, and then, dilutions series were prepared in a 96-well plate, ranging from 50 μM to 3000 μM. The antibacterial activity of the common standard antibiotic ampicillin (Merck Millipore, Darmstadt, Germany) and the antifungal fluconazole (Thermo Fisher Scientific, Cleveland, OH, USA) was also recorded using the same procedure as described above at concentrations ranging from 1 to 500 μM. Four bacteria strains were cultured with tested compounds for 24 h at 37 °C in Mueller–Hinton broth (MHB) (Merck Millipore, Darmstadt, Germany), while *C. albicans* ATCC 10231 was grown aerobically at 37 °C on Yeast Peptone Dextrose (YPD) broth (A&A Biotechnology, Gdańsk, Poland) in a 96 well plate. Growth control wells contain 1% DMSO. The optical density of each well was measured at 600 nm using a 96-well microplate reader (TECAN Spark 10 M; Tecan Group Ltd., Männedorf, Switzerland). The MIC was defined as the lowest concentration of the tested agents at which no microbial growth occurred. The minimal bactericidal concentration (MBC) and minimum fungicidal concentration (MFC) were determined by subculturing 10 μL of the medium collected from the wells containing compounds of concentrations equal to or greater than the MIC, on the Mueller–Hinton (for bacteria) and YPD (for yeast) agar plates. MBC and MFC values were defined as the lowest concentration of the tested compounds that reduced the number of CFU by 99.9% in comparison with the control. All the measurements were performed in three independent experiments.

### Microscopic Evaluation of Bacterial Membrane Permeabilization

*S. aureus* ATCC 25923 harvested in the log phase were incubated in MHB medium with **4** and **5**. Bacteria cells incubated without POSS derivatives were used as the control. After 24 h at 37 °C, microorganisms were washed with sterile phosphate-buffered saline (PBS; pH = 7.2). Then, the bacterial cells were stained for 30 min in the dark at 37 °C with Live/Dead BacLight Viability Kit (L-7007, Invitrogen) prepared in PBS buffer. Cells were visualized using fluorescence microscopy (Axio Scope A1, Zeiss, Jena, Germany). Images were acquired and analyzed using Carl Zeiss ZEN 2.3 lite software for quantification of live and dead cells.

## Results and Discussion

### Synthesis and Characterization of 1–6 Derivatives

**Scheme 1:**
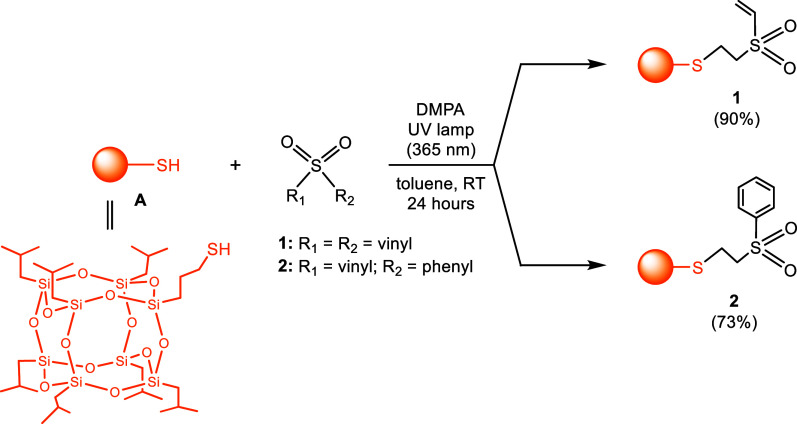
Synthesis of Sulfonyl Derivatives of Mono-POSS **1** and **2**

For the synthesis of sulfonyl and sulfonamide derivatives of cage-like silsesquioxanes, we utilized commercially available monosubstituted precursors (mono-POSS) – 3-mercaptoheptaisobutyl-POSS (precursor **A**) and 3-aminopropylheptaizobutyl-POSS (precursor **B**), respectively. As modifying agents for precursor **A**, divinyl and phenyl vinyl sulfones were used. The formation of sulfonyl derivatives occurred under mild conditions in toluene at room temperature, facilitated by DMPA as the photoinitiator of the thiol–ene reaction. To ensure the effectiveness of the DMPA, the reaction mixture was exposed to ultraviolet light with a wavelength of 365 nm for 24 h. The reaction yielded two sulfonyl derivatives, **1** and **2**. Derivative **1** features a vinyl group linked to the −SO_2_– fragment, while **2** incorporates a phenyl group, as shown in Scheme 1. The obtained species were characterized by spectroscopic methods such as nuclear magnetic resonance NMR (^1^H, ^13^C, and ^29^Si), Fourier transform infrared spectroscopy (FT-IR), and mass spectrometry (MALDI-MS), and their elemental composition was confirmed by elemental analysis (EA).

The aforementioned sulfone derivatives, containing monofunctionalized POSS entities **1** and **2**, were synthesized with high yields of 90% and 73%, respectively. The ^1^H NMR spectrum of **1** clearly exhibits chemical shifts for multiplets at 6.65, 6.45, and 6.18 ppm, indicative of the presence of a vinyl group (Figure S1). Detailed analysis via the ^13^C NMR spectrum (Figure S2) identifies methylene group peaks, components of the organic S-containing side chain attached to the inorganic silsesquioxane core, appearing at 54.62, 35.26, 24.06, 22.96, and 11.58 ppm. Chemical shifts in the ^29^Si NMR spectrum for **1** fall within the anticipated range for monosubstituted cubic POSS,^28,31^ recorded at −67.63, −67.86, and −68.24 ppm, which corroborates the presence of three distinct silicon nuclei configurations (Figure S3). For compound **2**, the ^1^H NMR spectrum (Figure S7) shows characteristic chemical shifts for phenyl groups at 7.90, 7.66, and 7.56 ppm. The ^13^C NMR spectrum (Figure S8) further elucidates the structure, with methylene groups and aromatic carbons noted at 56.52, 35.20, 24.26, 22.89, and 11.51 for −CH_2_– groups, and 138.94, 133.82, 129.39, 127.99 ppm for aromatic moiety. Similar to **1**, the ^29^Si NMR spectrum (Figure S9) of derivative **2** displays chemical shifts that validate the expected configuration for a monosubstituted POSS, at −67.65, −67.88, and −68.93 ppm, affirming the cage-like silsesquioxane structure with three types of silicon atoms. For both derivatives **1** and **2**, the mass spectra (Figures S5 and S11) of {MALDI-MS for **1**: *m*/*z*: 1031.28 (calcd [M + Na]^+^) 1031.28; and for **2**: *m/z:* 1081.29 (calcd [M + Na]^+^) 1081.28} conclusively verified a closed-frame architecture comprising eight silicon atoms. Seven silicon atoms are each surrounded by isobutyl groups, while one silicon nucleus is linked to a terminal side chain that incorporates a sulfonyl group. In the infrared spectra of **1** and **2** (Figures S4 and S10), broad bands indicating specific vibrations for terminal groups are observed at 743 (ν_S–C_) and 1327 (δ_S–O_) cm^–1^ for **1**, and at 736 (ν_S–C_) and 1368 (δ_S–O_) cm^–1^ for **2**. The vibrations at 2955 (ν_C–H/sym_) and 2928 (ν_C–H/asym_) cm^–1^ are associated with methylene groups in **1**, and at 2950 (ν_C–H/sym_) and 2927 (ν_C–H/asym_) cm^–1^ in **2**. Additionally, the Si–C bond vibrations are noted at 1231 and 1230 cm^–1^ for **1** and **2**, respectively. Furthermore, the absorption band at 1634 cm^–1^ is distinctly attributed to the ν_(C=C)_ mode for **1**. For sulfone derivative **2**, characteristic C–H aromatic vibrations are noted at 839 cm^–1^. For both derivatives, the absorption band characteristic of siloxane Si–O–Si bond vibrations appears narrow and strong, registering at 1110 and 1083 cm^–1^ for **1** and **2**, respectively.

Surprisingly, in the case of the thermal stability of derivatives **1** and **2** compared to precursor **A**, no significant differences were observed (Table S38). This can be explained by the fact that sulfone functionalities generally contribute to thermal stability due to their strong S=O bonds. However, if these groups are present in low concentrations relative to the silsesquioxane core, then their stabilizing effect might not be pronounced enough to cause a significant difference in degradation temperature. Additionally, if the degradation of **1** and **2** follows a pathway similar to that of precursor **A**—where the thermal breakdown occurs predominantly via the cleavage of siloxane bonds—then the overall thermal stability would remain nearly unchanged.

**Scheme 2:**
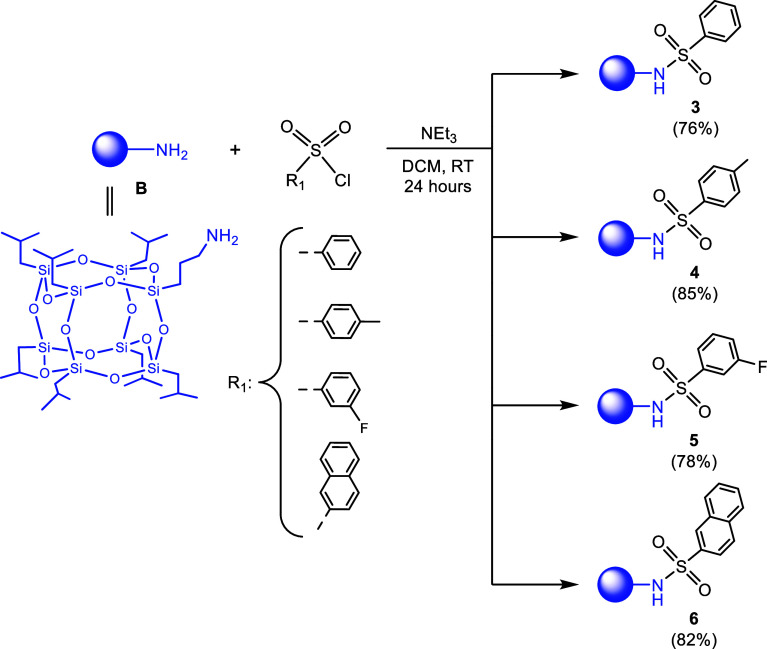
Synthesis of Sulfonamide Derivatives of Mono-POSS **3**-**6**

Sulfonamide derivatives of cage-like silsesquioxanes were synthesized in DCM through the reaction of precursor **B** with appropriate sulfonyl chlorides, such as benzenesulfonyl chloride, *p*-toluenesulfonyl chloride, 3-fluorobenzenesulfonyl chloride, and 2-naphthalenesulfonyl chloride (Scheme 2). All reactions were conducted under an inert atmosphere (dinitrogen) at room temperature in the presence of triethylamine. The sulfonamide-containing products **3–6** were successfully obtained with high yields ranging from 76 to 85%, and can be stored in air.

The sulfonyl chlorides were selected to diversify the chemical nature of the substituents attached to the sulfonic sulfur atom. Each substituent imparts unique electronic and steric properties. Additionally, the choice of modifying groups can be further justified by their potential impact on the antibacterial, antifungal, and other bioactive properties of the compounds. Thus, the chosen substituents can substantially change the biological activity of sulfonamide derivatives through their influence on the interactions with biological targets. For example, groups like benzenesulfonyl might enhance lipophilicity, thereby improving cell membrane penetration, while others like 3-fluorobenzenesulfonyl might increase binding affinity to specific enzymes or receptors due to their electronic characteristics. Incorporating substituents such as *p*-toluenesulfonyl and 2-naphthalenesulfonyl can lead to compounds with enhanced or specific activity against certain bacterial strains or fungi, contributing to a broader spectrum of antimicrobial activity.^38,39^

Compounds **3–6** were characterized by using NMR spectroscopy, FT-IR, MALDI-MS spectrometry, and elemental analysis. The ^1^H NMR spectra of compounds **3–5** display chemical shifts in the range typical for aryl groups, with specific values of 7.84, 7.55, and 7.49 ppm for **3** (Figure S13), 7.71 and 7.27 ppm for **4** (Figure S19), and 7.63, 7.54, 7.47, and 7.26 ppm for **5** (Figure S25). Additionally, a chemical shift at 2.38 ppm in the case of **4** identifies a methyl group, confirming it as a substituent on an aromatic ring in the para position. For compound **6** (Figure S32), the chemical shifts observed at 8.41, 7.95, 7.88, 7.81, and 7.61 ppm are characteristic for a naphthyl group. Across derivatives **3–6**, the ^1^H NMR spectra reveal chemical shifts typical of an –NH group at 4.31 ppm for **3**, 4.33 ppm for **4**, 4.39 ppm for **5**, and 4.40 ppm for **6**, with low integration values due to rapid proton exchange with the solvent’s deuterium (in this case, CDCl_3_). Additionally, analysis of the ^13^C NMR spectra (Figures S14, S20, S26, and S33) reveals benzyl group shifts for **3** at 140.17, 132.53, 129.06, and 126.96 ppm; 3-methylbenzyl group shifts for **4** at 143.24, 137.24, 129.65, 127.07, and 30.90 ppm; 2-fluorobenzyl group shifts for **5** at 163.51, 161.45, 142.40, 130.89, 122.70, 119.64, and 114.51 ppm; and naphthyl group shifts for **6** at 136.96, 134.79, 129.44, 128.73, 128.38, 127.90, and 122.32 ppm. The cage-like structure of monosubstituted silsesquioxanes **3–6** has been unequivocally confirmed using ^29^Si NMR spectroscopy (Figures S15, S21, S28 and S34). The spectra for all derivatives exhibit three characteristic chemical shifts typical of this type of system,^28,31^ −67.60, −67.87, −68.21 ppm for compound **3**, −67.60, −67.87, −68.17 ppm for **4**, −67.58, −67.86, −68.29 ppm for **5**, and −67.60, −67.88, −68.22 ppm for **6**. For compound **5**, the ^19^F NMR spectrum shows only one shift, observed at −109.52 ppm, which indicates that there is one fluorine atom in the 3-fluorobenzenesulfonyl fragment (Figure S27). Additionally, the structures were verified using MALDI-MS spectrometry, where peaks at *m*/*z*: 1036.28 {calcd [M + Na]^+^ 1036.30}, 1050.31 {calcd [M + Na]^+^ 1050.32} and 1066.28 {calcd [M + K]^+^ 1066.42}, 1054.28 {calcd [M + Na]^+^ 1054.30}, and 1086.31 {calcd [M + Na]^+^ 1086.32} correspond to the structure of derivatives **3**, **4**, **5**, and **6**, respectively (Figures S17, S23, S30 and S36). Moreover, in the infrared spectra of compounds **3–6** (Figures S16, S22, S29 and S35), broad absorption bands are observed, characteristic of specific vibration modes for sulfonamides. These bands occur at 3266 cm^–1^ for ν(N–H) and 1328 cm^–1^ for δ(S–O) in **3**, 3260 cm^–1^ for ν(N–H) and 1328 cm^–1^ for δ(S–O) in **4**, 3266 cm^–1^ for ν(S–C) and 1334 cm^–1^ for δ(S–O) in **5**, and 3258 cm^–1^ for ν(S–C) and 1328 cm^–1^ for δ(S–O) in **6**. Additionally, the vibrations at 2955 cm^–1^ for ν(C–H) (symmetric) and 2869 cm^–1^ for ν(C–H) (asymmetric) are linked to methylene groups in species **3**, **4**, and **5**, while for **6**, these occur at 2955 cm^–1^ (ν_C–H/sym_) and 2875 cm^–1^ (ν_C–H/asym_). Characteristic aromatic C–H deformation vibrations are noted at 833 cm^–1^ for compounds **3**, **4**, and **5**, and at 839 cm^–1^ for **6**. For all these derivatives, the absorption band indicative of a siloxane Si–O–Si bond is narrow and strong, appearing at 1086, 1086, 1081, and 1090 cm^–1^ for **3**, **4**, **5**, and **6**, respectively. Due to the presence of the siloxane band, the expected band for the C–F bond in **6**, typically visible in the range of 1200–1000 cm^–1^, is not observed.

**Figure 1:**
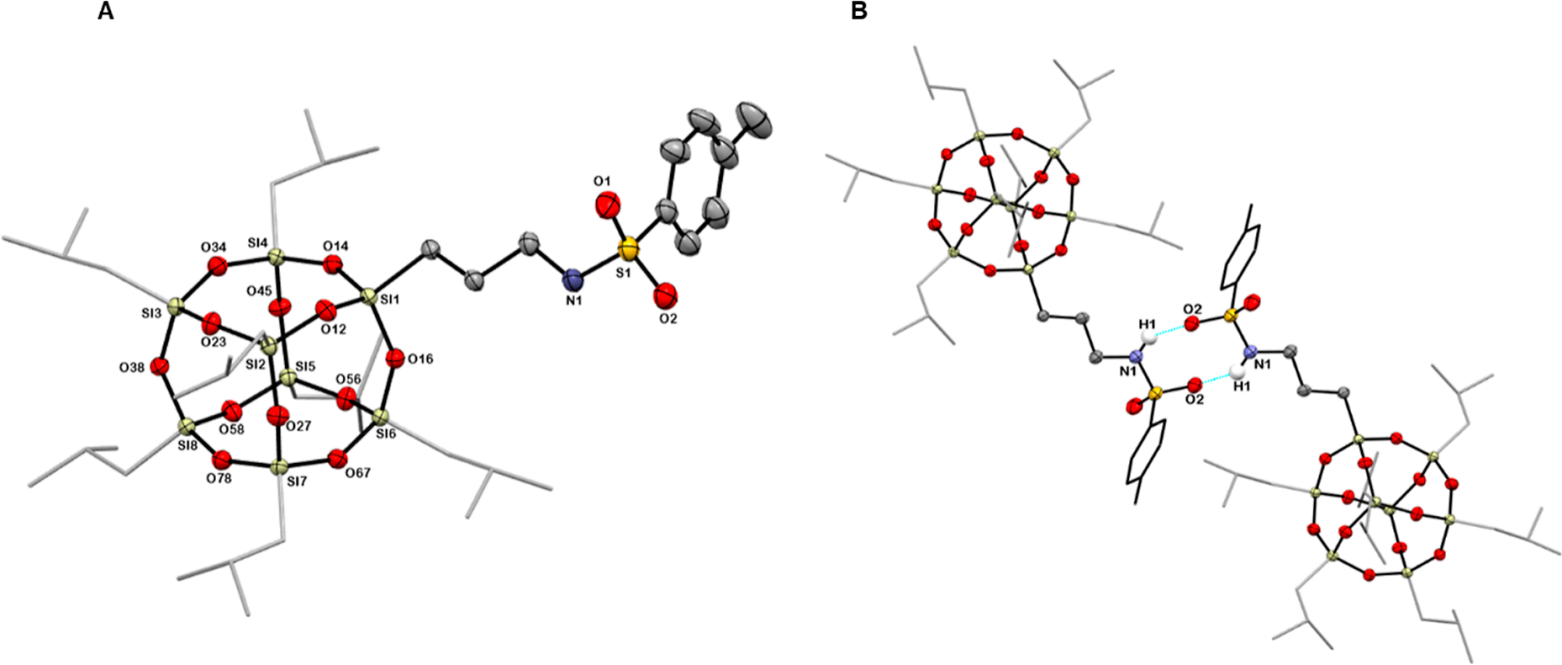
Molecular structure of **4** (A). Hydrogen bonds between the NH group and the oxygen atom of the sulfonamide group (B).

Additionally, single crystals were obtained for derivative **4**, which underwent X-ray structural analysis. The single-crystal X-ray diffraction revealed that compound **4**, depicted in Figure 1A, crystallizes in the triclinic system with the space group *P*1̅. Selected crystallographic data for the obtained crystal are listed in Table 1.

Compound **4** consists of eight silicon atoms connected by 12 oxygen atoms, forming the POSS cage core. It is a derivative of monosubstituted POSS, featuring seven isobutyl groups and one arm terminated with a tosyl group. Selected bond lengths and angles are provided in Table S1. **4** represents the first described in the literature crystalline structure of a sulfonamide POSS derivative, making it necessary to compare the crystal parameters of compound **4** only with those of compounds containing similar fragments, i.e., a sulfonamide group or a monosubstituted T_8_ cage. All bond lengths and angles of both the T_8_ cage and the sulfonamide group are consistent with the literature data. The S=O bond lengths in **4** range from 1.431 to 1.442 Å, matching those reported for sulfonamides by M. Le Bars,^40^ L. Li,^41^ and A. J. Lough.^42^ Similarly, the S–N and S–C bond lengths, measuring 1.614 and 1.767 Å, respectively, align with literature ranges of 1.58–1.62 and 1.76–1.79 Å. For the silicon–oxygen T_8_ cage of **4**, Si–O bond lengths are in the range of 1.617–1.626 Å, comparable to those observed in previously reported mono-POSS crystal structures, where Si–O bond lengths range from 1.607 Å to 1.643 Å.^43,44^

Furthermore, the crystal structure of **4** features intermolecular hydrogen bonds between the NH group and the oxygen atom of the sulfonamide group (Table 2). These hydrogen bonds further stabilize the packing of the crystal structure (Figure 1B).

**Table 2:** Hydrogen Bonds of **4**

*D*—H···*A*	*D*—H (Å)	H···*A* (Å)	*D*···*A* (Å)	*D*—H···*A* (°)
N1—H1···O2^i^	0.88	2.40	2.991(4)	125.3

Symmetry code(s): (i) −*x* + 2, −*y* + 1, −*z* + 1.

Thermogravimetric analysis (Figure S39) was performed for compounds **3–6** and their 3-aminopropylheptaisobutyl-POSS substrate (precursor **B**). The aim of this study was to examine the impact of differences in the structure of the side chain anchored to the silsesquioxane core on the thermal stability of the obtained monosubstituted derivatives. The measurement data are summarized in Table 3.

**Table 3:** Average 5% and 10% Mass Loss Temperatures for **3-6** Derivatives

compound	Δ*T*_5%_ (°C)	Δ*T*_10%_ (°C)
precursor **B**	292.97	315.81
**3**	326.37	344.95
**4**	333.84	358.54
**5**	207.99	225.70
**6**	339.80	378.37

For a mass loss of 5% and 10%, the thermal degradation temperature (Δ*T*_5%_ and Δ*T*_10%_, respectively) increased with the number of carbon atoms in the side organic arm, indicating enhanced thermal stability of the obtained derivatives compared to precursor **B**. A particularly interesting observation is the exceptionally low thermal stability of derivative **5**, which is surprising given that the CF bond energy significantly exceeds that of the C–C bond. The cause of this phenomenon may be attributed to the shift in the electron density of the ring toward the fluorine atom.

### Antimicrobial Activity

Creating antimicrobial surface coatings is crucial for combating hospital-acquired infections and microbial resistance to traditional treatments. Properly designed surfaces can effectively inhibit bacterial growth and activity. Given our understanding of antimicrobial resistance mechanisms, efforts are ongoing to counteract these challenges,^45^ for example, the development of coatings that hinder bacterial adhesion has led to the modification of various surfaces with multifunctional sulfonamide groups.^32^

Pathogen-induced infections can result in both acute and chronic illnesses. Common multidrug-resistant pathogens responsible for hospital-acquired infections include Gram-positive bacteria such as *S. aureus* and *E. hirae*, as well as Gram-negative bacteria like *E. coli* and *P. aeruginosa*. Gram-negative bacteria are generally more resistant to antibiotics due to their outer membrane, which serves as a permeability barrier.^46^ Moreover, projections indicate that by 2050, antimicrobial resistance could result in 10 million deaths annually, with healthcare costs rising to €1.5 billion per year.^47^ Consequently, there is an urgent need to develop new antimicrobial treatments.

Antimicrobial activities of tested compounds were investigated against a range of opportunistic human pathogens, including *E. coli* ATCC 25922, *P. aeruginosa* ATCC 15422, *E. hirae* ATCC 10541, *S. aureus* ATCC 25923, and fungus *C. albicans* ATCC 10231. Their MICs and minimum bactericidal/fungicidal concentrations (MBCs/MFCs) were determined using the serial broth microdilution method.^48^ As shown in Table 4, all tested compounds display high potency against pathogenic bacteria and *C. albicans* species, with MICs in the 125–3000 μM range and MBCs/MFCs that are 1–2 times higher than respective MICs (Table 5). In addition, the inhibitory effect was found to be stronger against bacteria *E. hirae* and *S. aureus*, as compared to Gram-negative bacteria *E. coli*, *P. aeruginosa,* and yeast *C. albicans*. We note that **5** outperformed other sulfonyl and sulfonamide derivatives in antimicrobial activities, although the difference was rather insignificant.

**Table 4:** MIC (μM) of Tested Agents, against Tested Bacteria and *Candida* Strains^a^

compounds	*E. coli* ATCC 25922 MIC (μM)	*P. aeruginosa* ATCC 15422 MIC (μM)	*E. hirae* ATCC 10541 MIC (μM)	*S. aureus* ATCC 25923 MIC (μM)	*C. albicans* ATCC 10231 MIC (μM)
divinyl sulfone	1500 ± 17	1500 ± 15	1500 ± 13	1500 ± 14	750 ± 6
**1**	500 ± 8	500 ± 13	250 ± 11	250 ± 21	500 ± 2
phenyl vinyl sulfone	1500 ± 11	3000 ± 17	3000 ± 12	1500 ± 16	750 ± 22
**2**	500 ± 10	1000 ± 12	250 ± 14	250 ± 7	500 ± 17
benzenesulfonyl chloride	1500 ± 12	1500 ± 15	750 ± 11	750 ± 11	1500 ± 37
**3**	500 ± 8	500 ± 5	250 ± 8	250 ± 4	500 ± 5
*p*-toluenesulfonyl chloride	1500 ± 16	3000 ± 21	750 ± 5	750 ± 6	3000 ± 14
**4**	500 ± 13	500 ± 8	250 ± 2	125 ± 4	500 ± 7
3-fluorobenzenesulfonyl chloride	1500 ± 10	1500 ± 10	375 ± 5	500 ± 13	3000 ± 34
**5**	250 ± 7	500 ± 12	125 ± 2	125 ± 17	500 ± 22
2-naphthalenesulfonyl chloride	3000 ± 17	3000 ± 16	1500 ± 13	1500 ± 15	750 ± 15
**6**	1000 ± 16	500 ± 15	250 ± 15	250 ± 8	500 ± 11
ampicillin	62.5 ± 4	n/d	31.25 ± 4	62.5 ± 2	
fluconazole					4 ± 0.5

a Results represent the averages of triplicate experiments ±SD. n/d, not determined within the concentration range used in this study.

**Table 5:** MBC (μM) and MFC (μM) of Tested Agents, against Tested Bacteria and *Candida* Strains^a^

compounds	*E. coli* ATCC 25922 MBC (μM)	*P. aeruginosa* ATCC15422 MBC (μM)	*E. hirae* ATCC 10541 MBC (μM)	*S. aureus* ATCC 25923 MBC (μM)	*C. albicans* ATCC 10231 MFC (μM)
divinyl sulfone	3000 ± 11	3000 ± 18	3000 ± 22	3000 ± 21	750 ± 11
**1**	500 ± 3	1000 ± 17	500 ± 14	500 ± 6	500 ± 12
phenyl vinyl sulfone	1500 ± 14	>3000 ± 25	>3000 ± 25	3000 ± 17	750 ± 7
**2**	1000 ± 7	>1000 ± 18	500 ± 9	500 ± 21	1000 ± 11
benzenesulfonyl chloride	3000 ± 23	3000 ± 11	1500 ± 19	1500 ± 15	3000 ± 23
**3**	1000 ± 14	1000 ± 21	500 ± 14	500 ± 17	1000 ± 5
*p*-toluenesulfonyl chloride	3000 ± 12	>3000 ± 19	1500 ± 16	1500 ± 11	>3000 ± 17
**4**	1000 ± 17	500 ± 8	500 ± 8	250 ± 12	1000 ± 22
3-fluorobenzenesulfonyl chloride	3000 ± 25	3000 ± 21	750 ± 11	1000 ± 21	>3000 ± 24
**5**	500 ± 11	1000 ± 6	250 ± 13	250 ± 14	1000 ± 7
2-naphthalenesulfonyl chloride	>3000 ± 24	>3000 ± 12	3000 ± 22	3000 ± 16	1500 ± 6
**6**	>1000 ± 11	500 ± 7	500 ± 8	500 ± 14	1000 ± 11
ampicillin	62.5 ± 4	n/d	31.25 ± 4	62.5 ± 2	
fluconazole					4 ± 0.5

a Results represent the averages of triplicate experiments ±SD. n/d, not determined within the concentration range used in this study.

**Figure 2:**
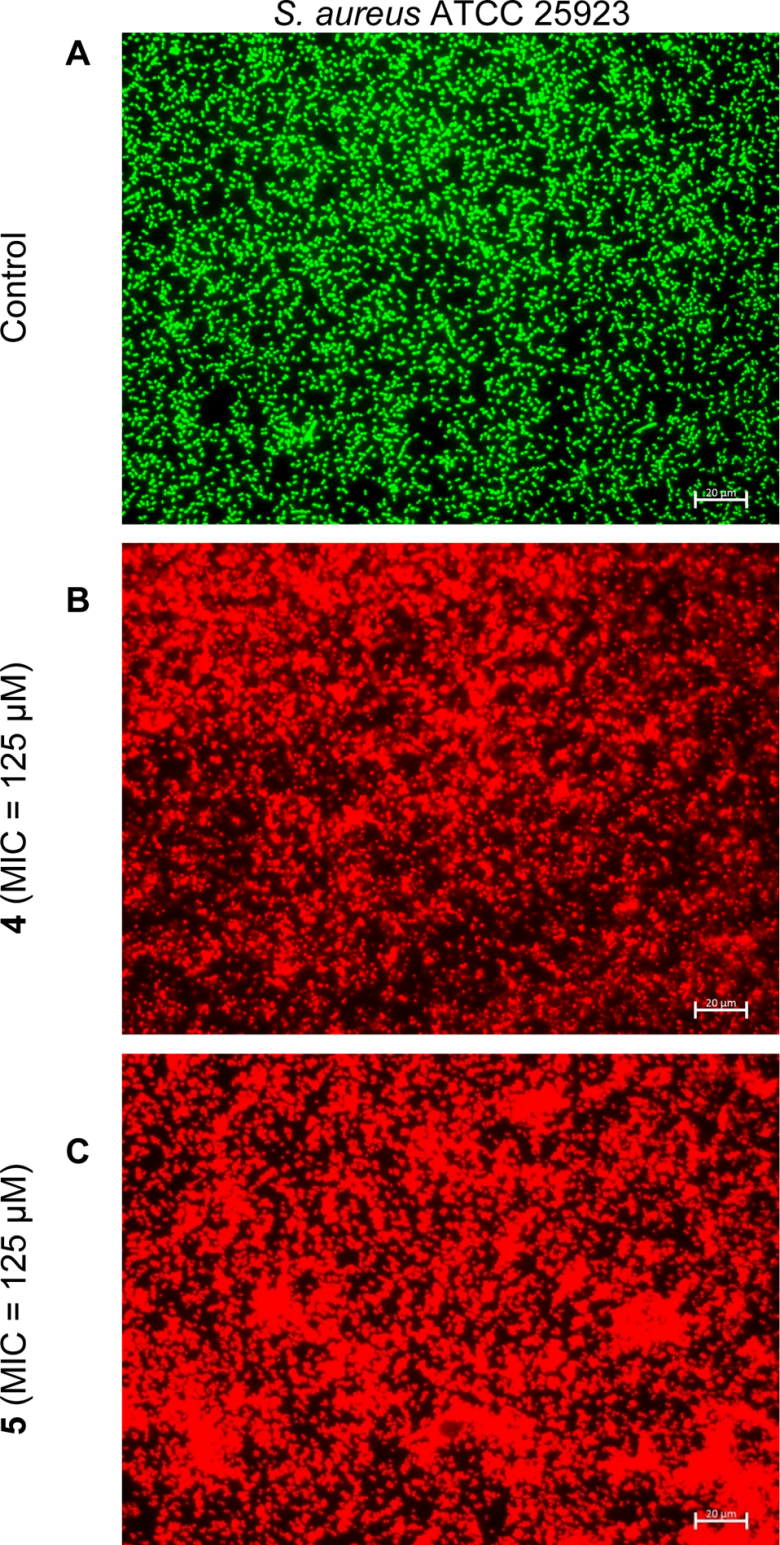
Fluorescence microscopy assays for the study of the viability of *S. aureus* treated with the **4** and **5** derivatives. (A) Bacteria in green indicate live/healthy cells, whereas (B,C) red are indicative of dead or membrane damaged bacteria. Scale bar −20 μm.

In this work, we hypothesized that the antimicrobial activity of POSS derivatives is achieved via interactions of these compounds with negatively charged microbial cells, leading to membrane disruption followed by lysis and cell death. Moreover, the increased lipophilic character favors interactions between POSS derivatives and lipids located in the cell membrane.^22^ Fluorescence microscopy was employed to further investigate the hypothesized membrane-disruption mechanism. Fluorescence microscopy, utilizing nucleic acid stains, such as SYTO-9 and PI, is a pivotal technique for assessing cell membrane integrity and elucidating antimicrobial mechanisms. SYTO-9 is a green-fluorescent dye that permeates both live and dead cells, binding to nucleic acids and emitting fluorescence upon excitation. In contrast, PI is a red-fluorescent dye that penetrates only cells with compromised membranes, intercalating into DNA and emitting fluorescence upon excitation. The combined use of these dyes enables differentiation between live and dead cells based on their fluorescence profiles. In the control assays (Figure 2A), mainly the green color is observed, which indicates a large number of live cells. The treatment with the POSS derivatives **4** and **5** (Figure 2B,C) led to a complete red color, which indicates dead cells. Similar effects were observed for the other tested compounds at their respective MIC values and across different pathogens, where membrane disruption and subsequent cell death were also evident (data not shown). While we hypothesize that membrane disruption is a primary mechanism of action for the antimicrobial activity of the tested compounds, we acknowledge that further molecular-level analyses, such as molecular dynamics simulations and lipophilicity assays, could provide additional insights into the interactions between these compounds and microbial membranes. Furthermore, we recognize that other mechanisms, including the generation of reactive oxygen species or enzyme inhibition, may contribute to the overall antimicrobial effect.^49^ Antimicrobial agents, in general, may also exert additional bactericidal effects, such as interfering with intracellular processes such as protein synthesis, DNA replication, or metabolic pathways, thereby enhancing their overall efficacy. While the evidence points to membrane damage as a primary mechanism, it is important to consider that POSS derivatives might also engage in other bactericidal mechanisms. Despite the clear indication of membrane disruption, these additional mechanisms remain of great interest and warrant further exploration in future studies.

*In vitro* research utilizing functionalized POSS molecules can contribute to the development of antibacterial coatings that are highly biocompatible and nontoxic. However, to ensure their application in clinical settings, these findings need to be extensively investigated and confirmed through clinical trials.

## Conclusions

This research highlights the innovative synthesis and significant antimicrobial efficacy of sulfone and sulfonamide derivatives incorporating POSS. The developed synthesis protocols are efficient, scalable, and yield high derivatives with potent antimicrobial properties, especially against Gram-positive bacteria, with MICs ranging from 125–3000 μM. Two sulfone derivatives (ethylvinylsulfone-POSS and phenylethylsulfone-POSS) and four sulfonamide derivatives (benzenesulfonamide-POSS, *p*-toluenesulfonamide-POSS, 3-fluorobenzenesulfonamide-POSS, and 2-naphthalenesulfonamide-POSS) were obtained with high yields (73–90%).

The unique cage-like architecture of POSS plays a pivotal role in our design. By providing a rigid and stable inorganic core, POSS enhances the durability of the hybrid materials and promotes efficient interaction with microbial membranes. The increased lipophilic character imparted by POSS facilitates membrane penetration, leading to an effective membrane disruption and subsequent cell lysis. This mechanism is especially pronounced against Gram-positive bacteria, which are known to be more susceptible to such interactions. This strategic incorporation significantly boosts antimicrobial efficacy compared to their non-POSS precursors, with derivative **5** (3-fluorobenzenesulfonamide-POSS) showing superior antimicrobial activity, likely due to its interaction with microbial membranes leading to cell disruption and death. The improved antimicrobial activity observed in our derivatives underscores the significance of incorporating POSS into these sulfur-based frameworks, a breakthrough that opens new avenues for the development of advanced antimicrobial coatings and therapeutic agents. Moreover, our work addresses the need for sustainable and cost-effective synthetic protocols in the field of hybrid material design. By employing straightforward, high-yield reactions under mild conditions, our method not only reduces synthetic complexity but also offers scalability for modern, higher-scale applications. This is particularly important, as the search for new antimicrobial agents intensifies in response to the growing threat of antibiotic resistance.

The findings underscore the potential of POSS-based derivatives as innovative solutions in medicinal chemistry, drug delivery systems, and materials science. They open new avenues for antimicrobial drug development, particularly in response to rising antibiotic resistance. Future research will focus on exploring the full spectrum of biological activities and optimizing functionalization processes to enhance the applicability and effectiveness of these promising compounds. Additionally, assessing their cytotoxicity will be crucial to evaluating their safety profile and ensuring their therapeutic potential. Clinical trials will provide further insights into their efficacy and real-world applicability as antimicrobial agents. Another important research direction will be investigating the antifungal mechanisms of these derivatives, particularly their potential against resistant *Candida* pathogens. These efforts will contribute to the development of new effective treatments for both bacterial and fungal infections, addressing the growing challenge of antimicrobial resistance.

## Supporting Information Available

^1^H, ^13^C, ^19^F, ^29^Si NMR, FT-IR, and MALDI-MS spectra of **1–6** and their elemental analyses, TGA analysis, and X-ray crystallographic data for **4** (PDF)
