# Supplementary material for: Enhanced Antimicrobial Efficacy of Sulfones and Sulfonamides via Cage-Like Silsesquioxane Incorporation
Source: Inorg Chem. 2025 Mar 25;64(13):6460–9. doi: 10.1021/acs.inorgchem.4c05156 (PMC11979884; doi:10.1021/acs.inorgchem.4c05156)
Supplement: Supplementary file 1 — ic4c05156_si_001.pdf [file ic4c05156_si_001.pdf]

Supporting Information

Enhanced Antimicrobial Efficacy of Sulfones and Sulfonamides *via* Cage-like Silsesquioxane Incorporation

Kamila Fuchs[a], Tomasz Janek[b], Mateusz Karpl[a], Anna Władyczyn[a], Jolanta Ejfler[a], Łukasz John[a,*]

[a] *Faculty of Chemistry, University of Wrocław, 14 F. Joliot-Curie, 50-383 Wrocław, Poland*

[b] *Department of Biotechnology and Food Microbiology, Wrocław University of Environmental and Life Sciences, 37 Chełmońskiego, 51-630 Wrocław, Poland*

*Corresponding author: Łukasz John, e-mail: lukasz.john@uwr.edu.pl

Table of Contents

1. Characterization of ethylvinylsulfone-POSS (1)

Figure S1. ^{1}H NMR (500 MHz, $CDCl_3$, 300 K) spectrum of **1**…………………...…… S4

Figure S2. ^{13}C NMR (126 MHz, $CDCl_3$, 300 K) spectrum of **1** ……………………. S4

Figure S3. ^{29}Si NMR (99 MHz, $CDCl_3$, 300 K) spectrum of **1** ………………..…… S5

Figure S4. FT-IR spectrum (KBr pellet) of **1** ……………………………………… S5

Figure S5, MALDI-MS ([M + Na]$^+$) of **1** …………………………………………. S6

Figure S6. Elemental Analysis of **1** ……………………………………………….... S6

2. Characterization of phenylethylsulfone-POSS (2)

Figure S7. ^{1}H NMR (500 MHz, $CDCl_3$, 300 K) spectrum of **2** …………………….. S7

Figure S8. ^{13}C NMR (126 MHz, $CDCl_3$, 300 K) spectrum of **2** ……………………. S7

Figure S9. ^{29}Si NMR (99 MHz, $CDCl_3$, 300 K) spectrum of **2** …………..……….... S8

Figure S10. IR spectrum of **2** ………………..…………………………………….. S8

Figure S11. MALDI-MS ([M + Na]$^+$) of **2** ……………………………………….. S9

Figure S12. Elemental Analysis of **2** ……………………………………………… S9

3. Characterization of benzenesulfonamid-POSS (3)

Figure S13. ^{1}H NMR (500 MHz, $CDCl_3$, 300 K) spectrum of **3** …………………….. S10

Figure S14. ^{13}C NMR (126 MHz, $CDCl_3$, 300 K) spectrum of **3** ……………………. S10

Figure S15. ^{29}Si NMR (99 MHz, $CDCl_3$, 300 K) spectrum of **3** …..………………… S11

Figure S16. IR spectrum of **3** ……………………………………………………… S11

Figure S17. MALDI-MS ($[M + Na]^+$) of **3** ……………………………………..…. S12

Figure S18. Elemental Analysis of **3**………………………………………………. S12

4. Characterization of *p*-toluenesulfonamid-POSS (4)

Figure S19. ^{1}H NMR (500 MHz, $CDCl_3$, 300 K) spectrum of **4** …………………….. S12

Figure S20. ^{13}C NMR (126 MHz, $CDCl_3$, 300 K) spectrum of **4** ……………………. S13

Figure S21. ^{29}Si NMR (99 MHz, $CDCl_3$, 300 K) spectrum of **4** …..………………… S13

Figure S22. IR spectrum of **4** ……………………………………………………… S14

Figure S23. MALDI-MS ($[M + Na]^+$, $[M + K^+]$) of **4** …………..………………….. S14

Figure S24. Elemental Analysis of **4** ………………………………………………… S14

Table S1. Selected Bond Lengths (Å) and Angles (°) of **4** ………………………... S15

5. Characterization of 3-fluorobenzenesulfonamid-POSS (5)

Figure S25. ^{1}H NMR (500 MHz, $CDCl_3$, 300 K) spectrum of **5** …………………….. S16

Figure S26. ^{13}C NMR (126 MHz, $CDCl_3$, 300 K) spectrum of **5** ……………………. S16

Figure S27. ^{19}F NMR (470 MHz, $CDCl_3$, 300 K) spectrum of **5** ……………………. S17

Figure S28. ^{29}Si NMR (99 MHz, $CDCl_3$, 300 K) spectrum of **5** ……….…………… S17

Figure S29. IR spectrum of **5** ……………………………………………………… S18

Figure S30. MALDI-MS ($[M + Na]^+$) of **5** …………………………………………. S18

Figure S31. Elemental Analysis of **5** ………………………………………………… S18

6. Characterization of 2-naphthalenesulfonamid-POSS (6)

Figure S32. ^{1}H NMR (500 MHz, $CDCl_3$, 300 K) spectrum of **6** …………………….. S19

Figure S33. ^{13}C NMR (126 MHz, $CDCl_3$, 300 K) spectrum of **6** ……………………. S19

Figure S34. ^{29}Si NMR (99 MHz, $CDCl_3$, 300 K) spectrum of **6** …..………………… S20

Figure S35. IR spectrum of **6** ……………………………………………………… S20

Figure S36. MALDI-MS ($[M + Na]^+$) of **6** ………………………………………….. S21

Figure S37. Elemental Analysis of **6** ………………………………………………… S21

7. Comparative thermogravimetric analysis (TGA)

Figure S38. Thermal gravimetric analysis (TGA) profiles of **1, 2** and **T$_8$iPrSH** …..... S22

Figure S39. Thermal gravimetric analysis (TGA) profiles of **3**, **4**, **5**, **6** and **T$_8$iPrNH$_2$** S22

8. The solubility of the resulting hybrids

Table S2. The solubility of **1-6** in common solvents …………………………….. S23

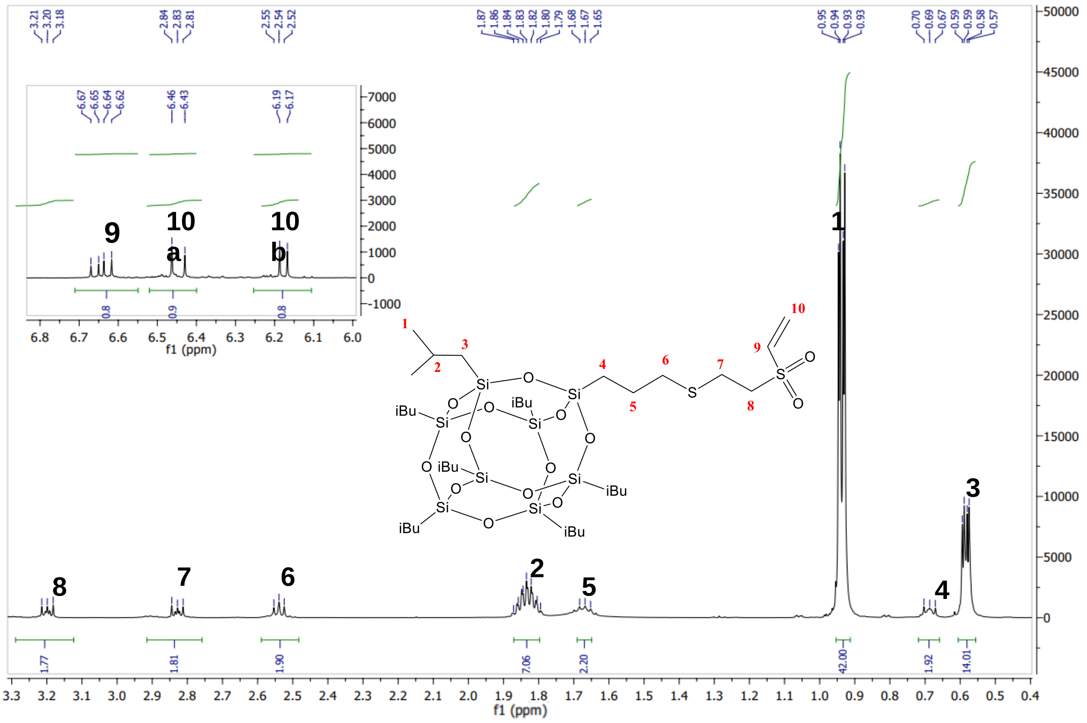

Figure S1. ^{1}H NMR (500 MHz, $CDCl_3$, 300 K) spectrum of **1**.

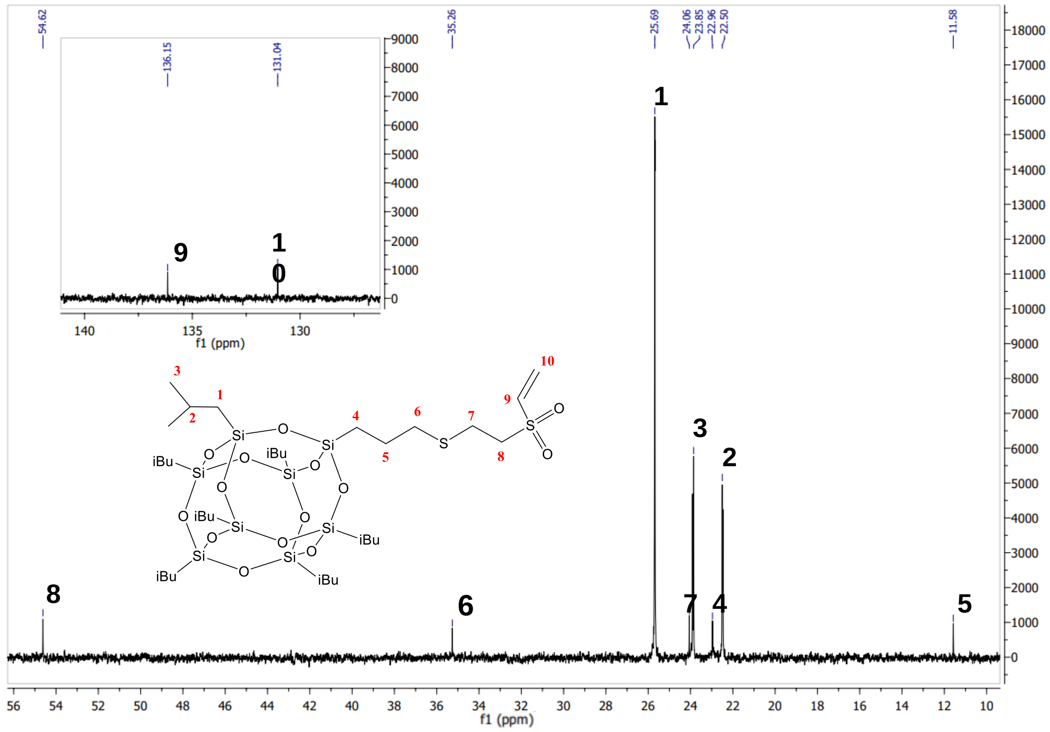

Figure S2. ^{13}C NMR (126 MHz, $CDCl_3$, 300 K) spectrum of **1**.

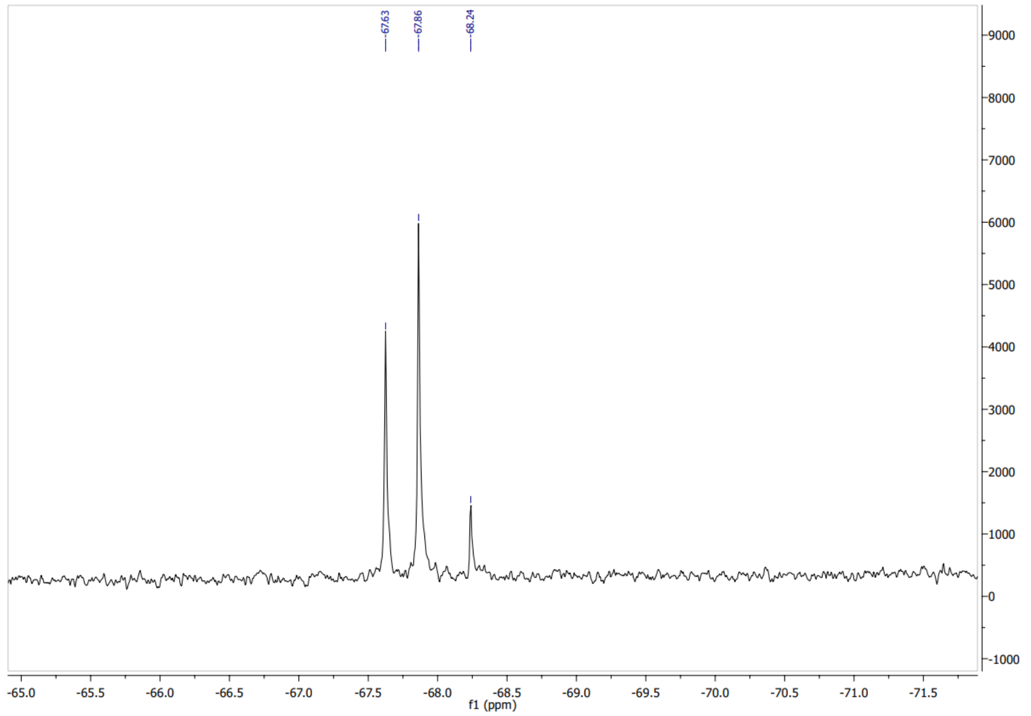

Figure S3. ^{29}Si NMR (99 MHz, $CDCl_3$, 300K) spectrum of **1**.

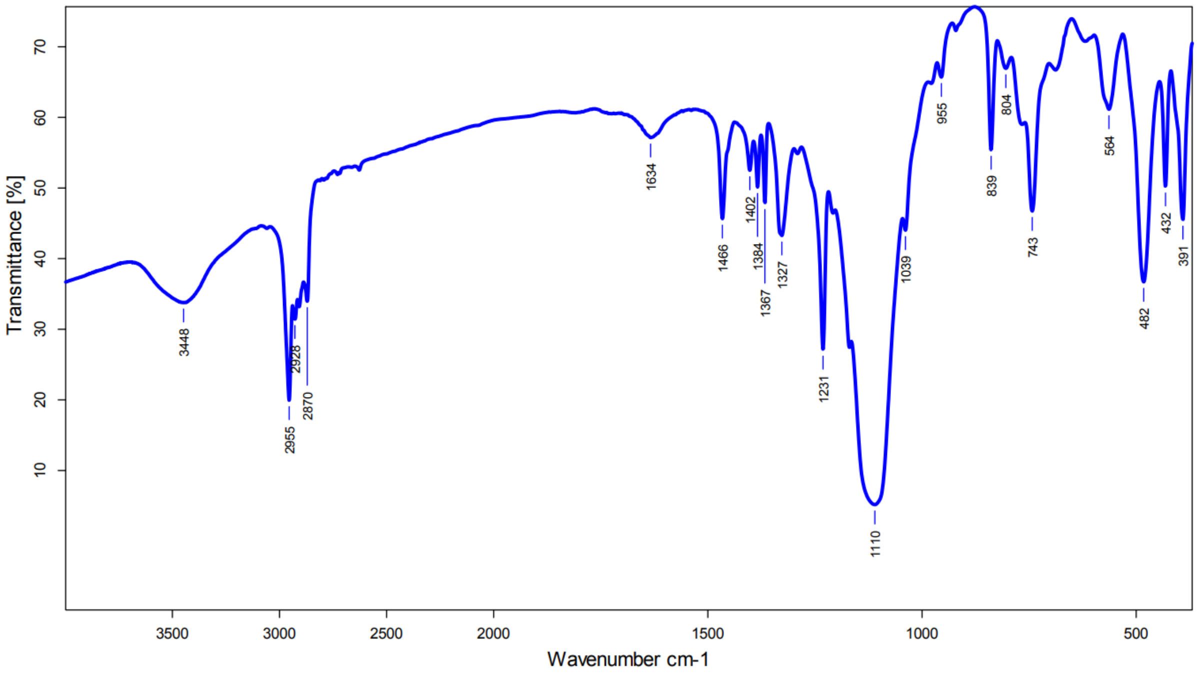

Figure S4. FT-IR spectrum (KBr pellet) of **1**.

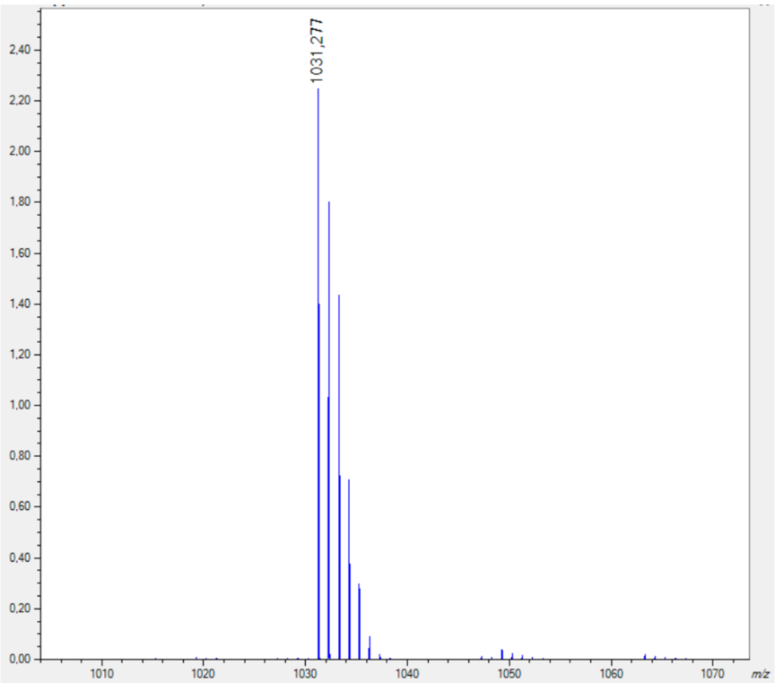

Figure S5. MALDI-MS ([M + Na]$^+$) of **1**.

	%C	%H	%S
Calculated	41.63	7.59	6.35
**Measured**	**41.43**	**7.53**	**6.23**

Figure S6. Elemental analysis of **1**.

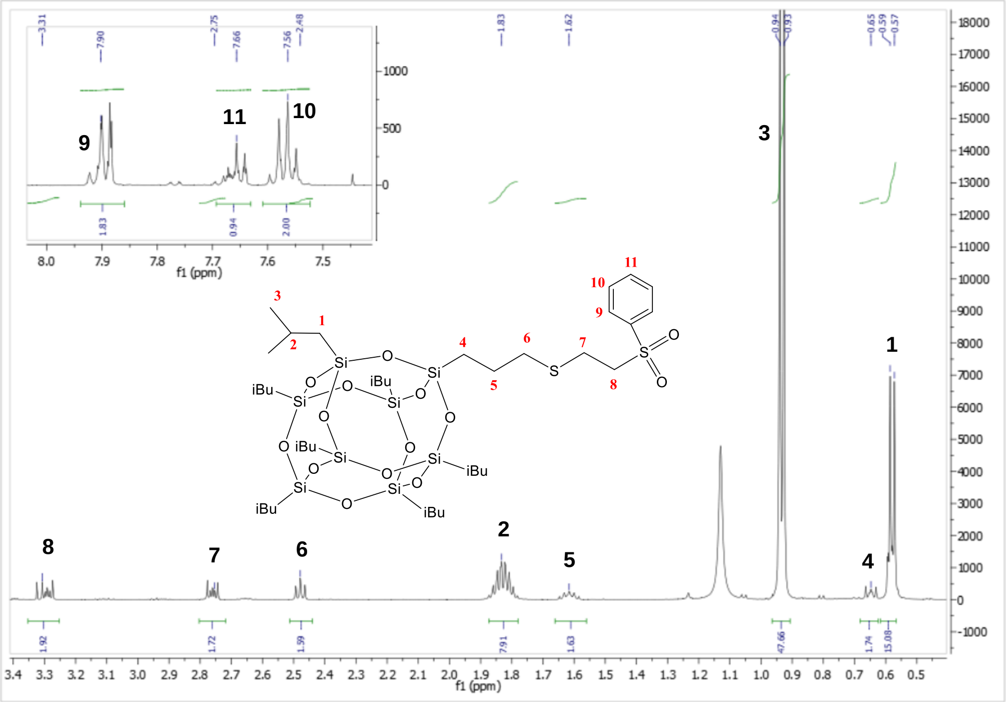

Figure S7. ^{1}H NMR (500 MHz, $CDCl_3$, 300 K) spectrum of **2**.

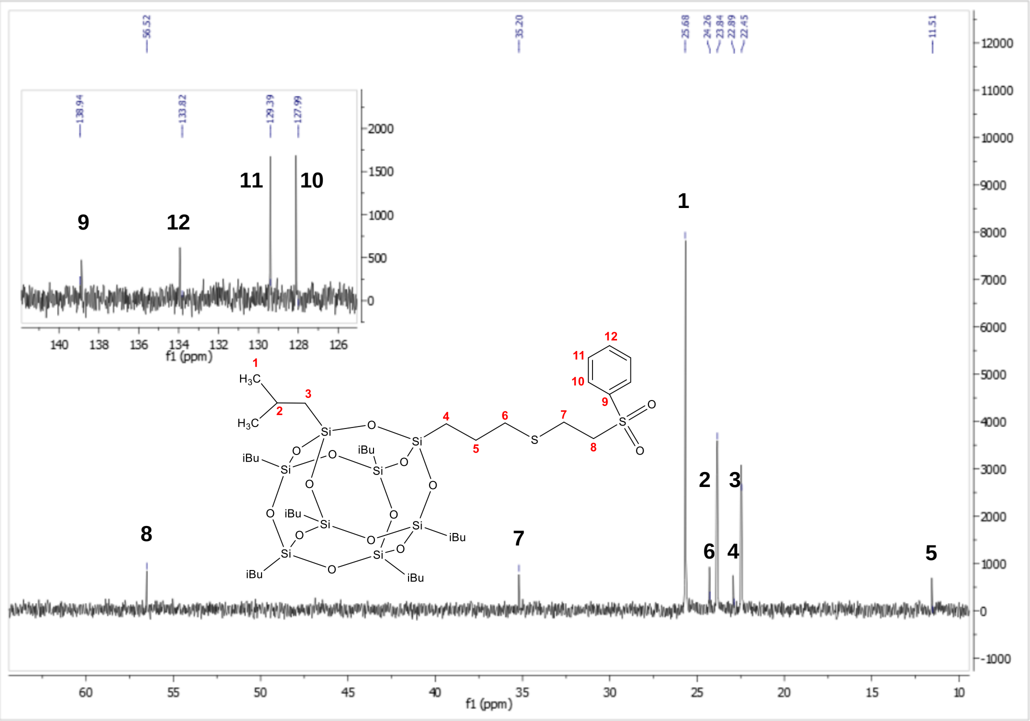

Figure S8. ^{13}C NMR (126 MHz, $CDCl_3$, 300 K) spectrum of **2**.

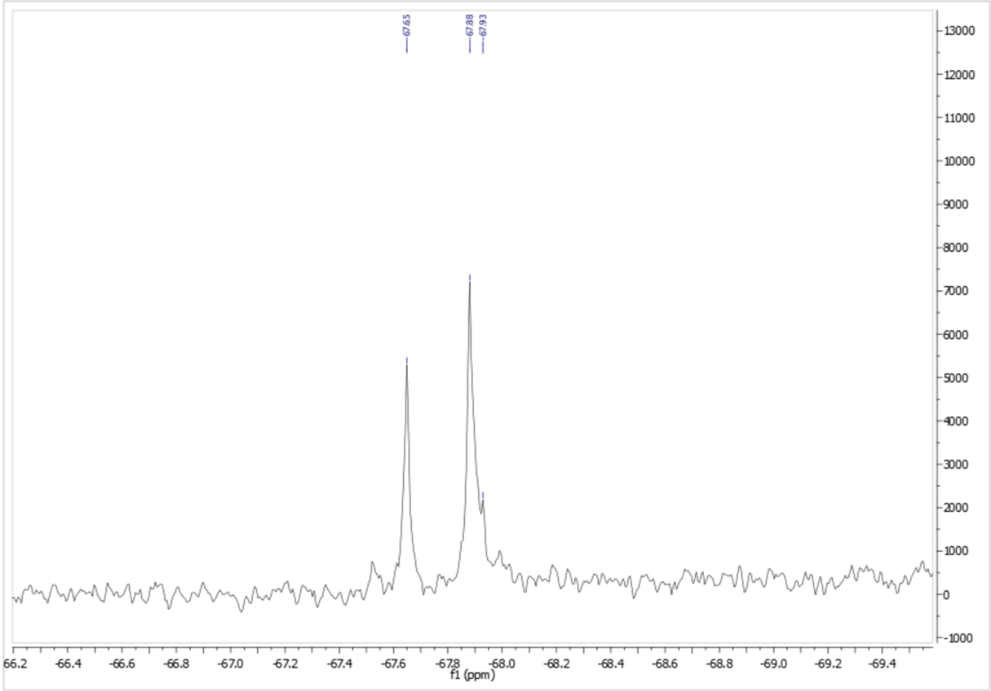

Figure S9. ^{29}Si NMR (99 MHz, $CDCl_3$, 300 K) spectrum of **2**.

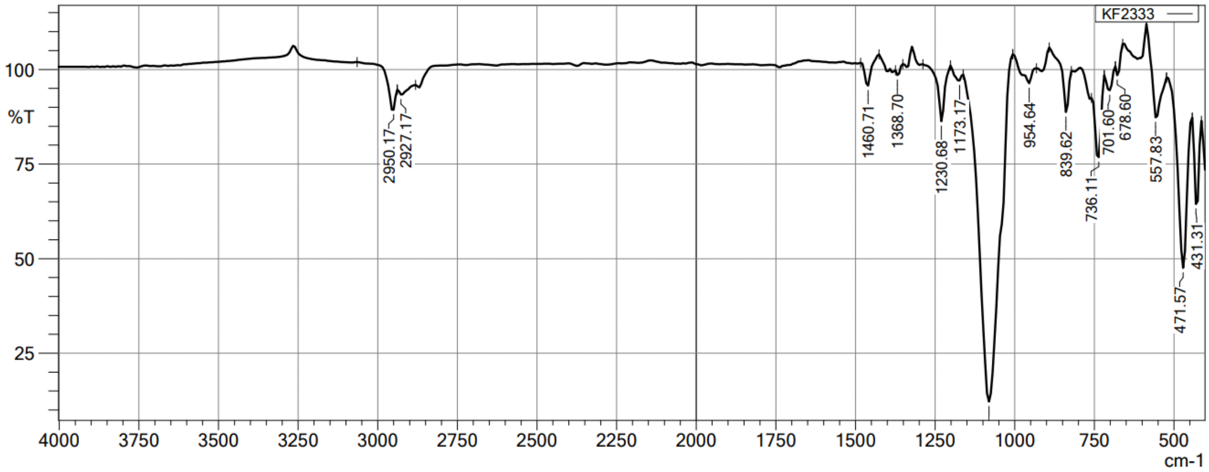

Figure S10. FT-IR spectrum of **2**.

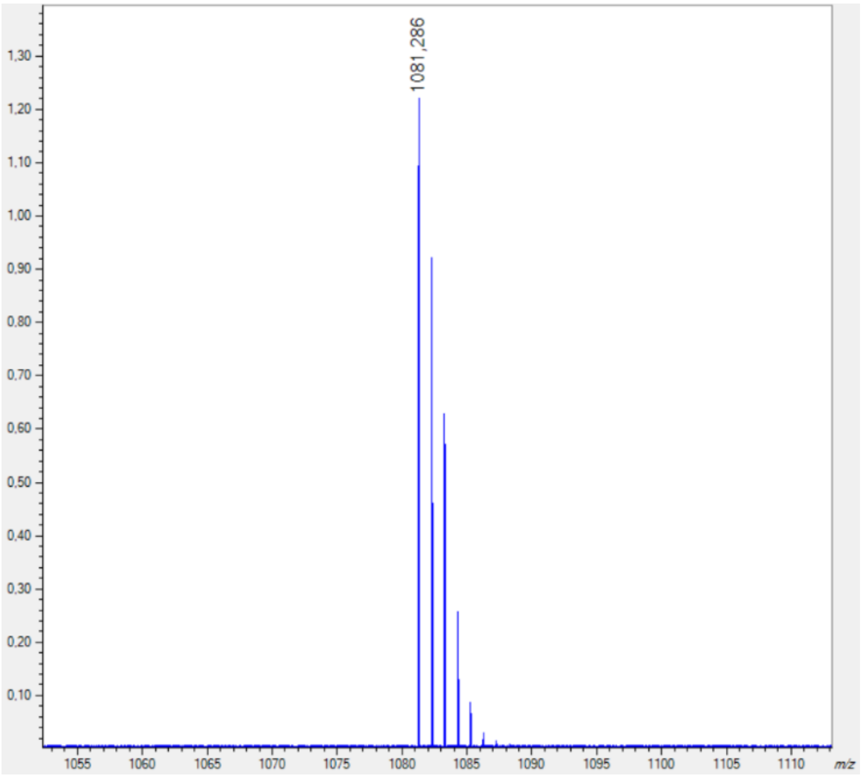

Figure S11. MALDI-MS ([M + Na]$^+$) of **2**.

	%C	%H	%S
Calculated	44.20	7.42	6.05
**Measured**	**44.03**	**7.31**	**5.89**

Figure S12. Elemental analysis of **2**.

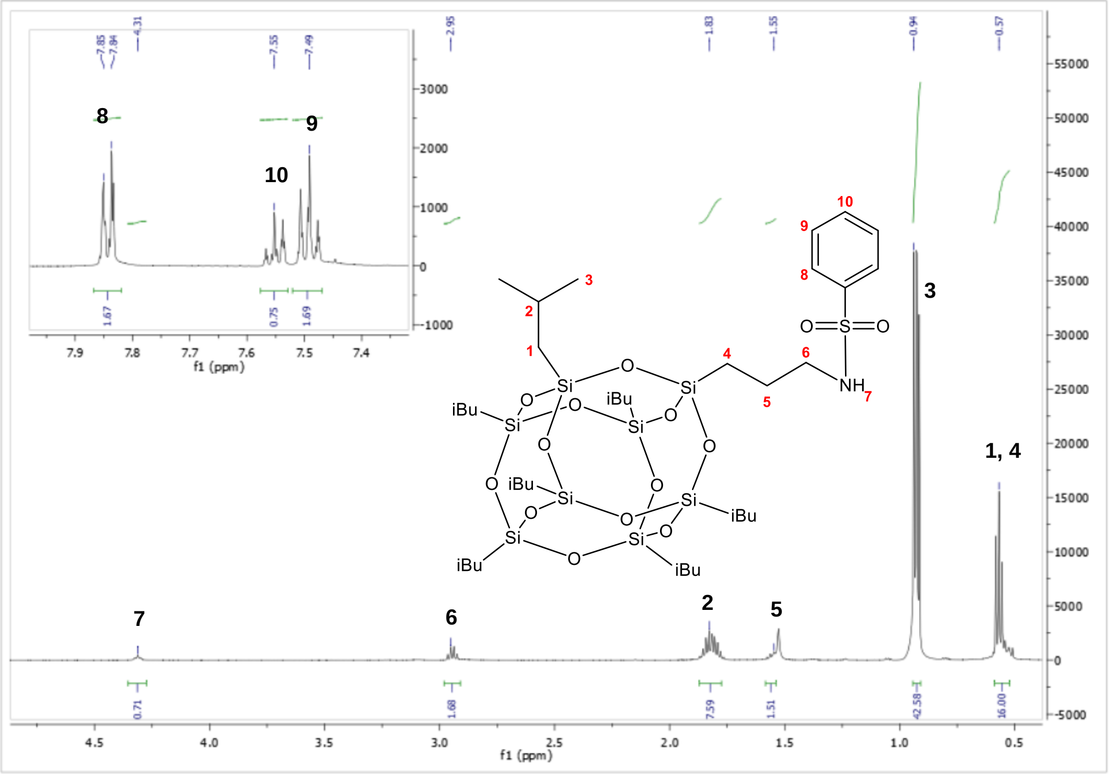

Figure S13. ^{1}H NMR (500 MHz, $CDCl_3$, 300 K) spectrum of **3**.

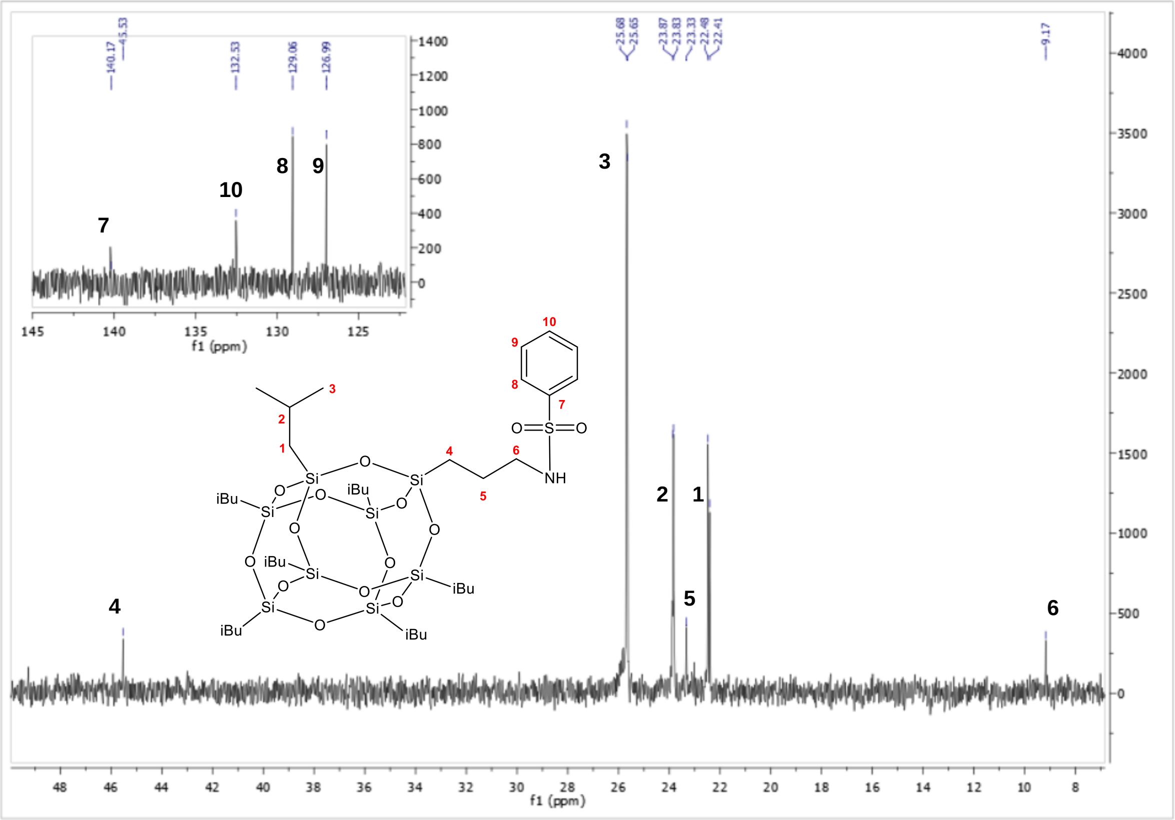

Figure S14. ^{13}C NMR (126 MHz, $CDCl_3$, 300 K) spectrum of **3**.

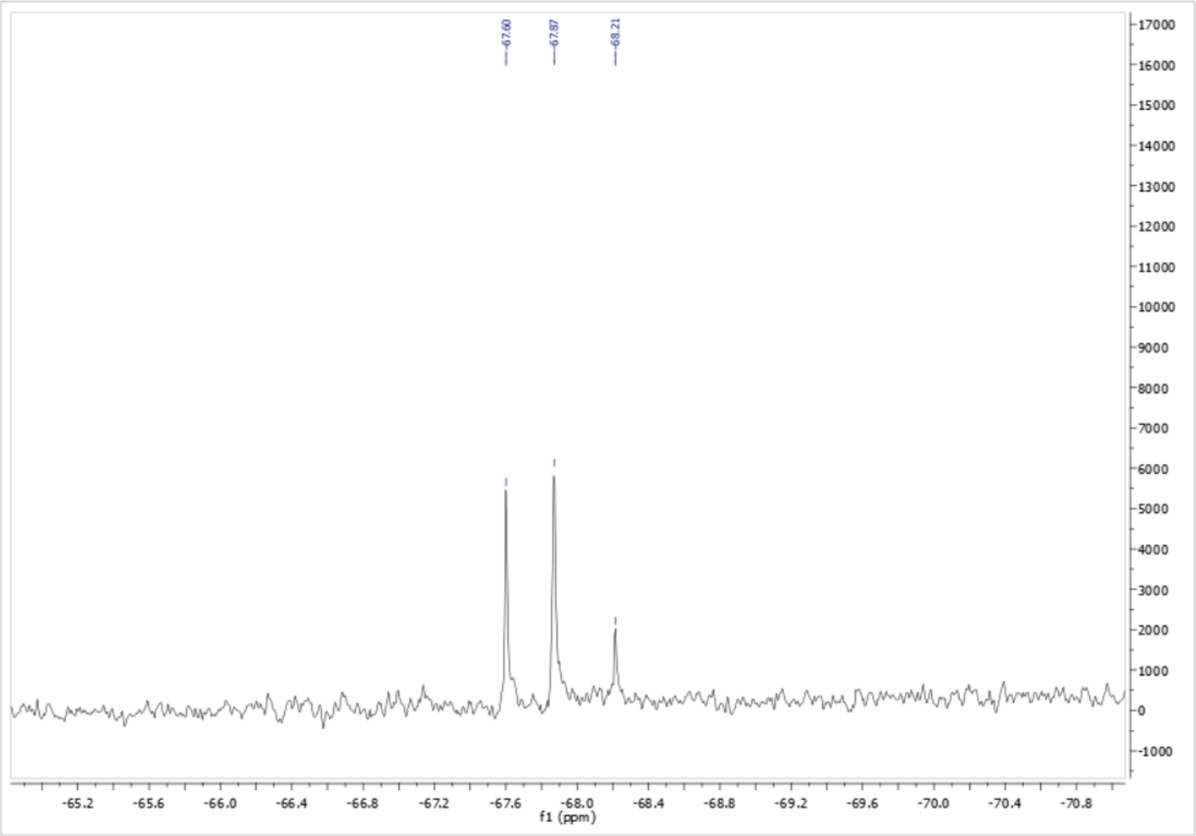

Figure S15. ^{29}Si NMR (99 MHz, $CDCl_3$, 300 K) spectrum of **3**.

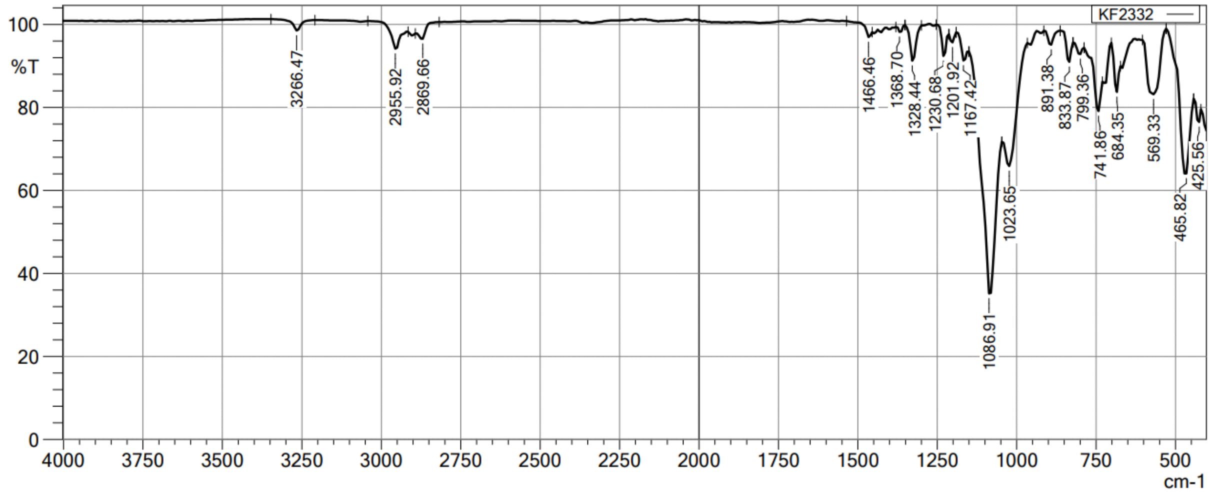

Figure S16. FT-IR spectrum of **3**.

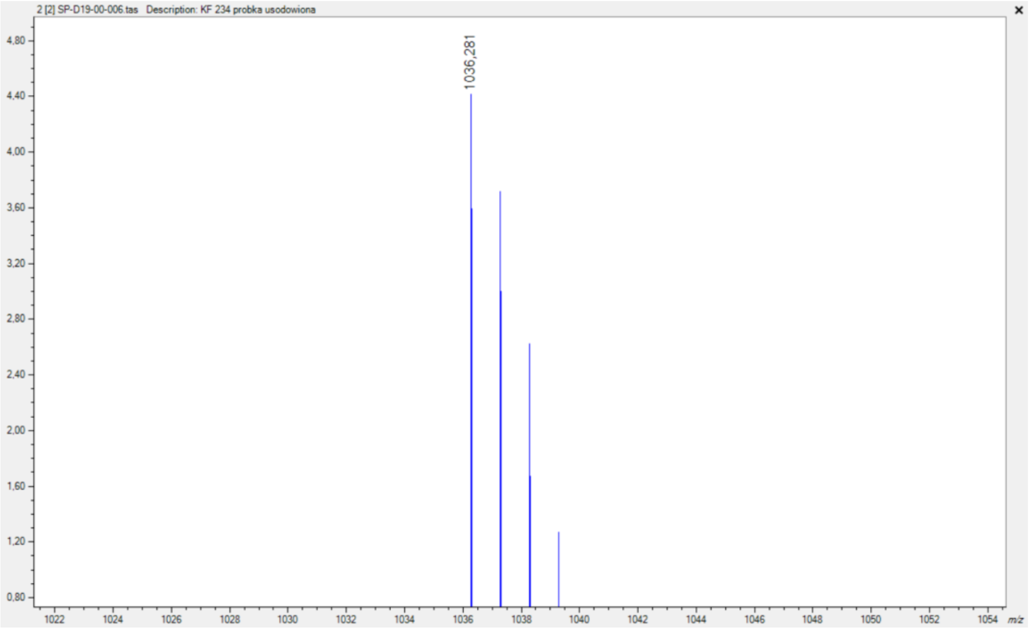

Figure S17. MALDI-MS ([M + Na]$^+$) of **3**.

	**%C**	**%H**	**%N**	**%S**
Calculated	43.80	7.45	1.38	3.16
**Measured**	**43.91**	**7.53**	**1.27**	**3.03**

Figure S18. Elemental analysis of **3**.

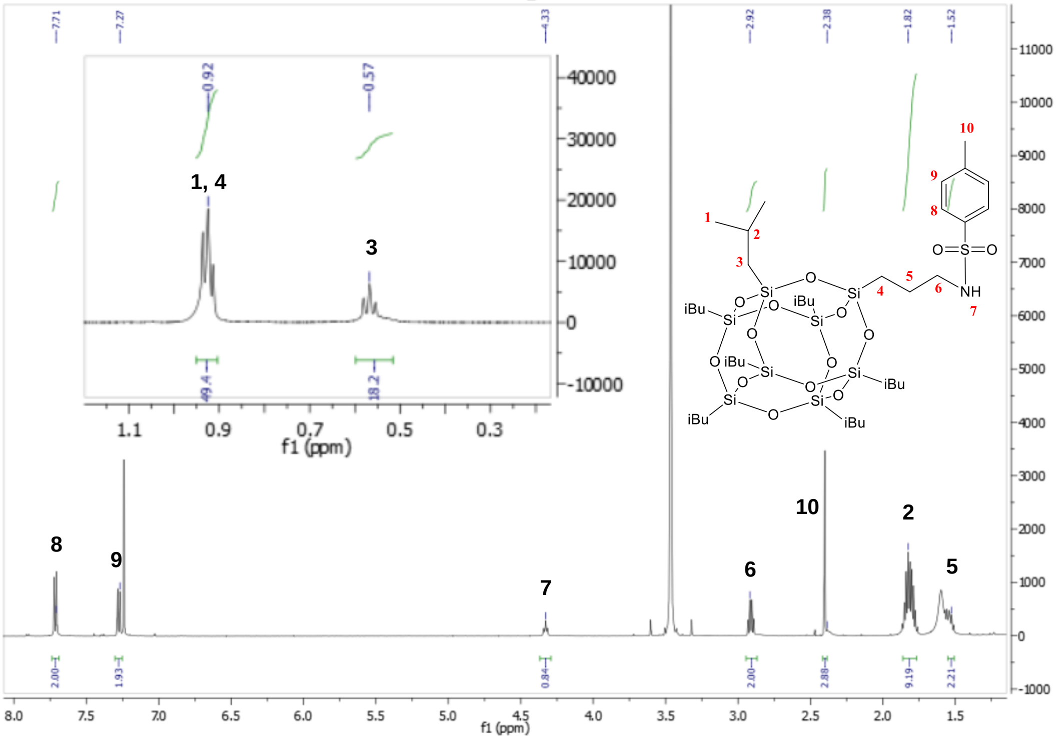

Figure S19. ^{1}H NMR (500 MHz, $CDCl_3$, 300 K) spectrum of **4**.

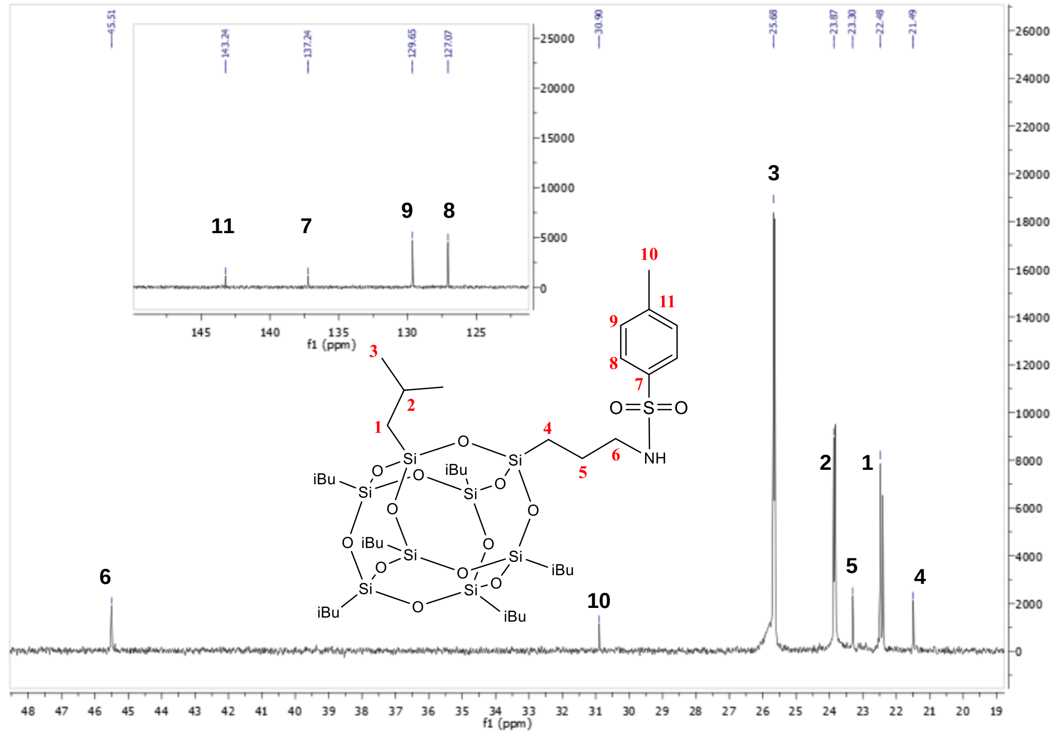

Figure S20. ^{13}C NMR (126 MHz, $CDCl_3$, 300 K) spectrum of **4**.

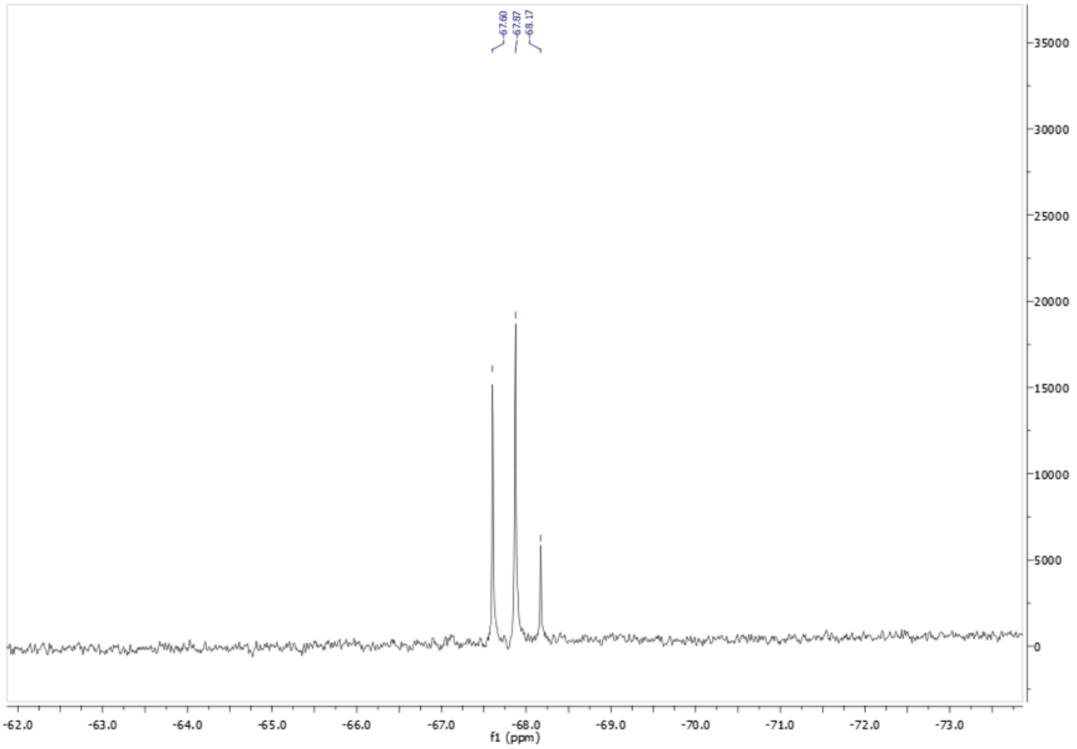

Figure S21. ^{29}Si NMR (99 MHz, $CDCl_3$, 300 K) spectrum of **4**.

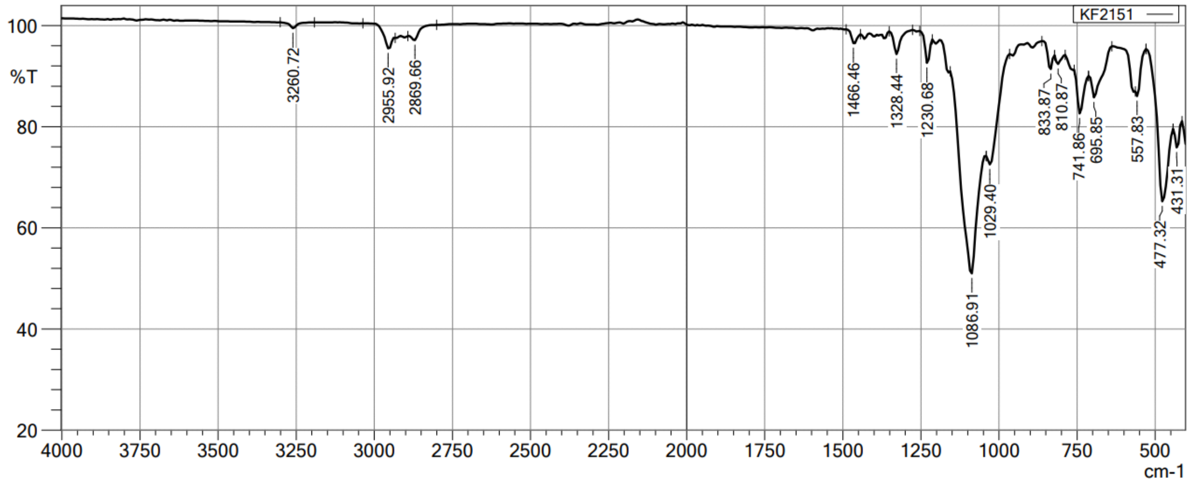

Figure S22. FT-IR spectrum of **4**.

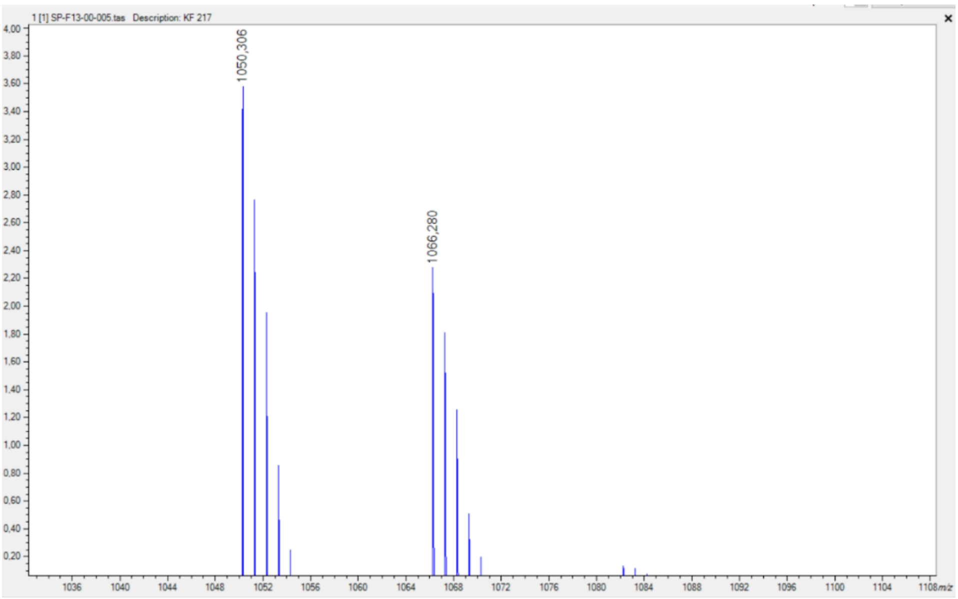

Figure S23. MALDI-MS ([M + Na]$^+$, [M + K$^+$]) of **4**.

	%C	%H	%N	%S
Calculated	44.37	7.54	1.36	3.12
**Measured**	**44.23**	**7.51**	**1.42**	**3.19**

Figure S24. Elemental analysis of **4**.

Table S1. Selected Bond Lengths (Å) and Angles (°) of **4**.

Si1—O14	1.617(2)	Si5—O45	1.626(2)
Si1—O12	1.621(2)	Si6—O56	1.617(2)
Si1—O16	1.626(2)	Si6—O16	1.621(2)
Si2—O23	1.619(2)	Si6—O67	1.622(2)
Si2—O27	1.622(2)	Si7—O67	1.620(2)
Si2—O12	1.626(2)	Si7—O78	1.621(2)
Si3—O23	1.619(2)	Si7—O27	1.626(2)
Si3—O34	1.621(2)	Si8—O58	1.618(2)
Si3—O38	1.625(2)	Si8—O78	1.619(2)
Si4—O34	1.620(2)	Si8—O38	1.618(19)
Si4—O14	1.620(2)	O1—S1	1.431(2)
Si4—O45	1.625(2)	O2—S1	1.442(2)
Si5—O58	1.617(2)	S1—N1	1.614(3)
Si5—O56	1.618(2)	S1—C4	1.767(4)
O14—Si1—O12	109.91(11)	O56—Si5—O45	108.75(11)
O14—Si1—O16	109.29(11)	O56—Si6—O16	109.17(11)
O12—Si1—O16	108.57(11)	O56—Si6—O67	108.52(11)
O23—Si2—O27	108.90(11)	O16—Si6—O67	109.02(11)
O23—Si2—O12	109.15(11)	O67—Si7—O78	109.11(11)
O27—Si2—O12	109.29(11)	O67—Si7—O27	109.07(11)
O23—Si3—O34	108.75(11)	O78—Si7—O27	108.70(11)
O23—Si3—O38	109.39(11)	O58—Si8—O78	109.03(11)
O34—Si3—O38	108.79(11)	O58—Si8—O38	109.17(11)
O34—Si4—O14	108.60(11)	O78—Si8—O38	108.72(11)
O34—Si4—O45	109.95(11)	O1—S1—O2	120.07(15)
O14—Si4—O45	108.35(10)	O1—S1—N1	108.43(14)
O58—Si5—O56	109.16(11)	O2—S1—N1	105.70(14)
O58—Si5—O45	108.76(11)	N1-S1-C4	106.95(15)

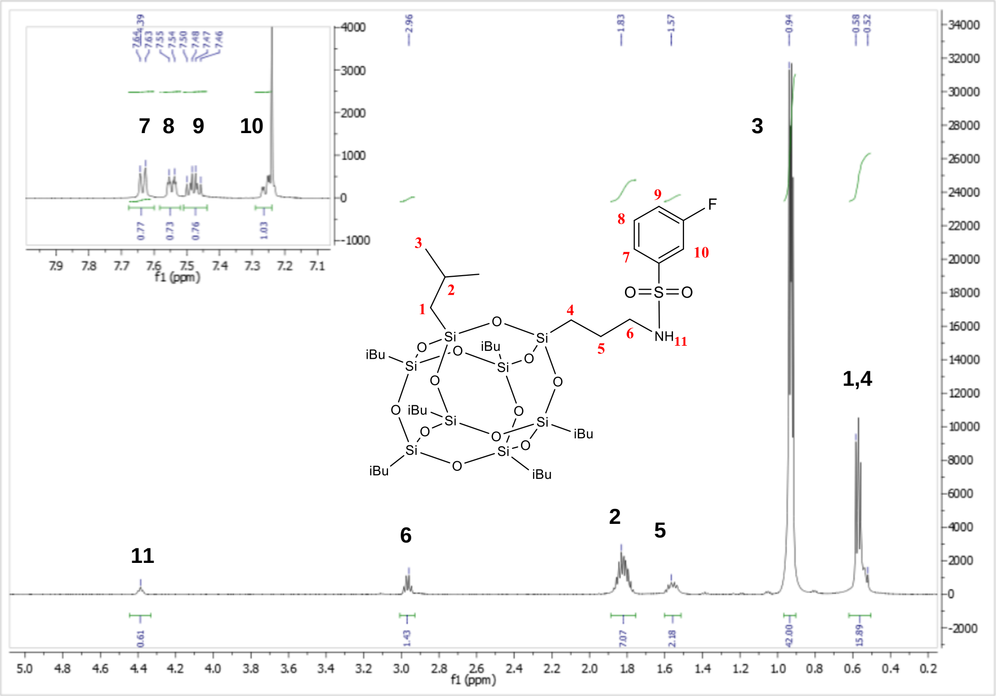

Figure S25. ^{1}H NMR (500 MHz, $CDCl_3$, 300 K) spectrum of **5**.

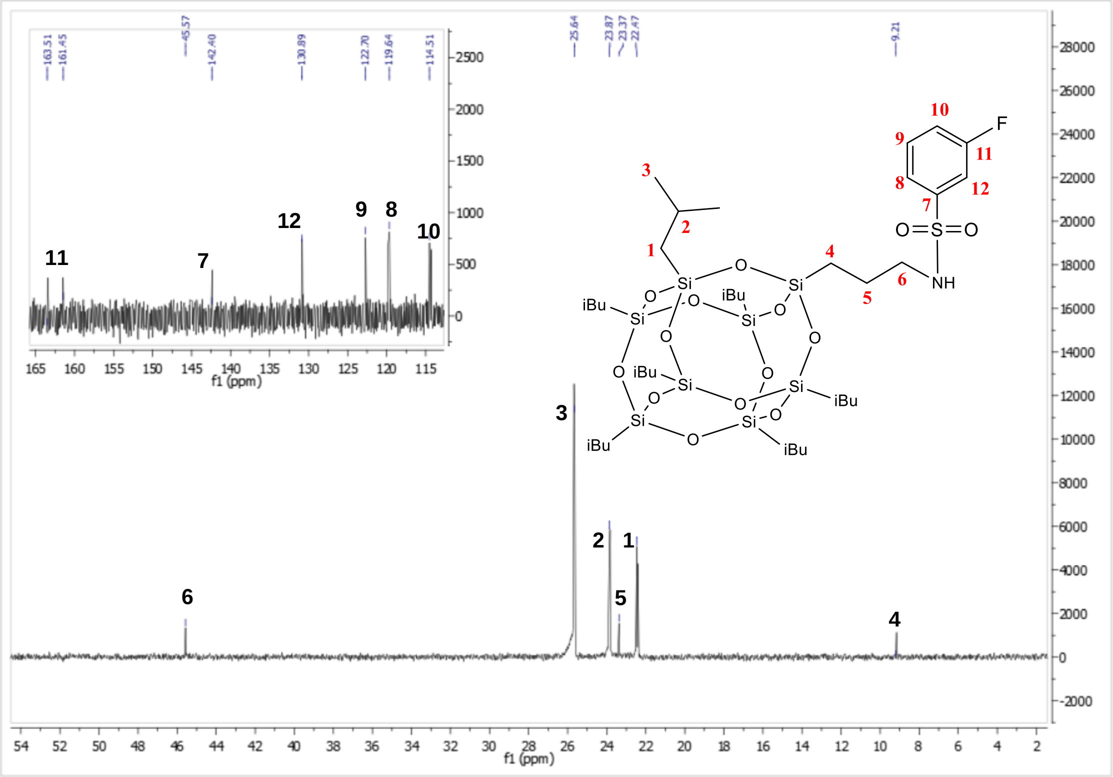

Figure S26. ^{13}C NMR (126 MHz, $CDCl_3$, 300 K) spectrum of **5**.

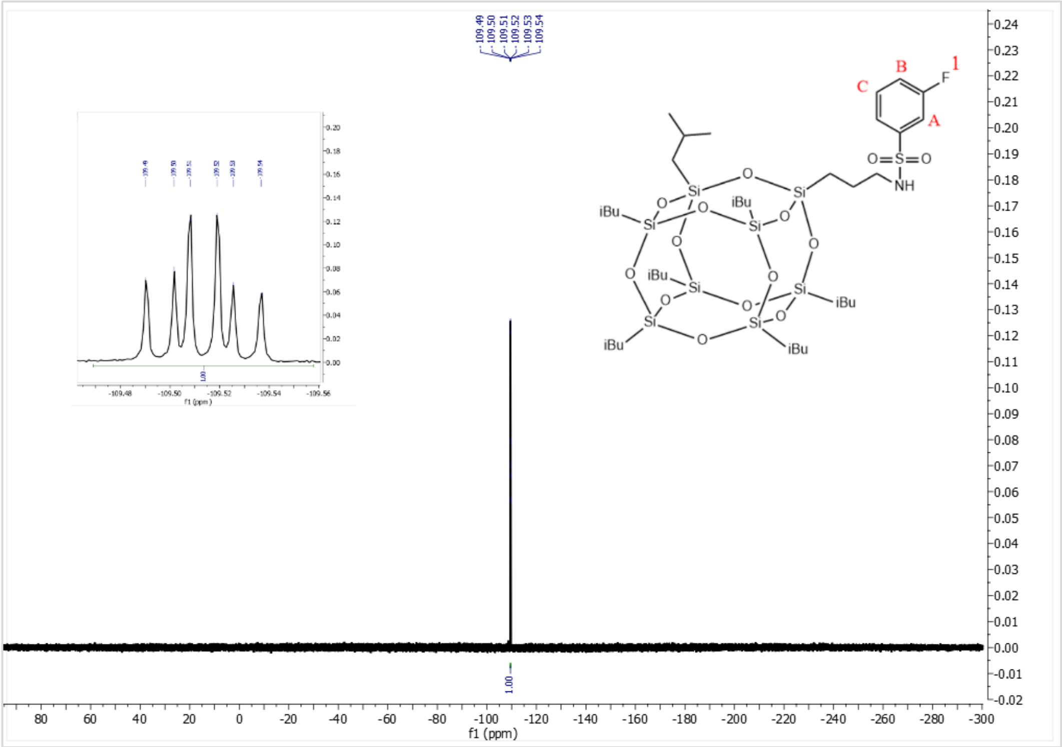

Figure S27. ^{19}F NMR (470 MHz, $CDCl_3$, 300 K) spectrum of **5**.

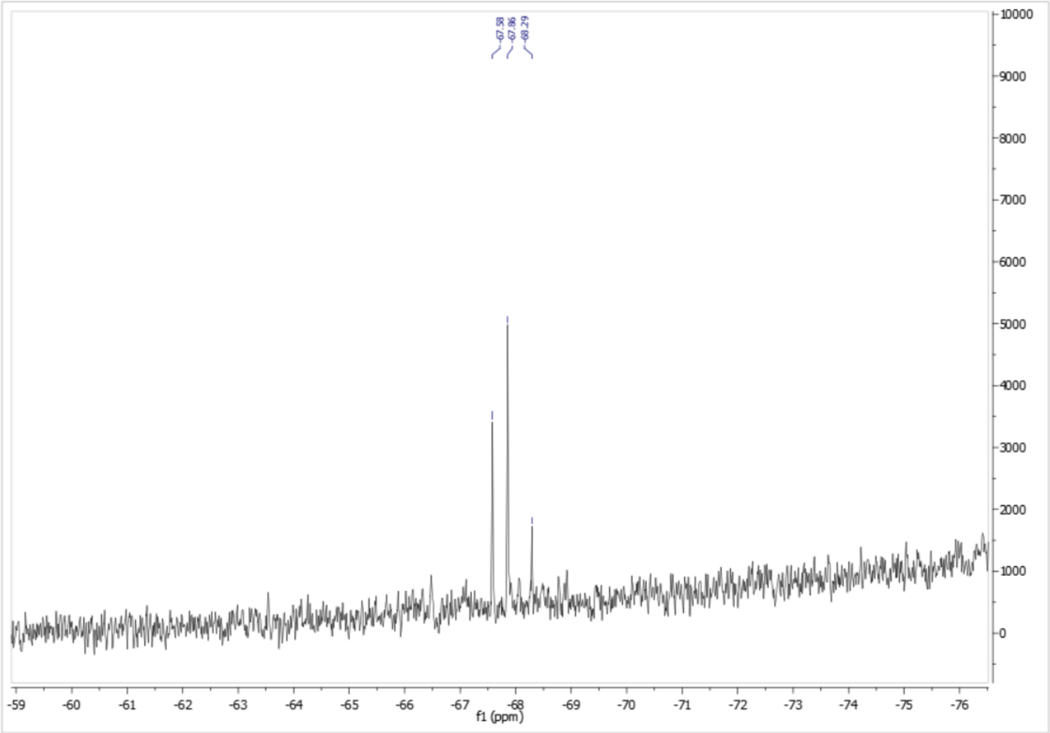

Figure S28. ^{29}Si NMR (99 MHz, $CDCl_3$, 300 K) spectrum of **5**.

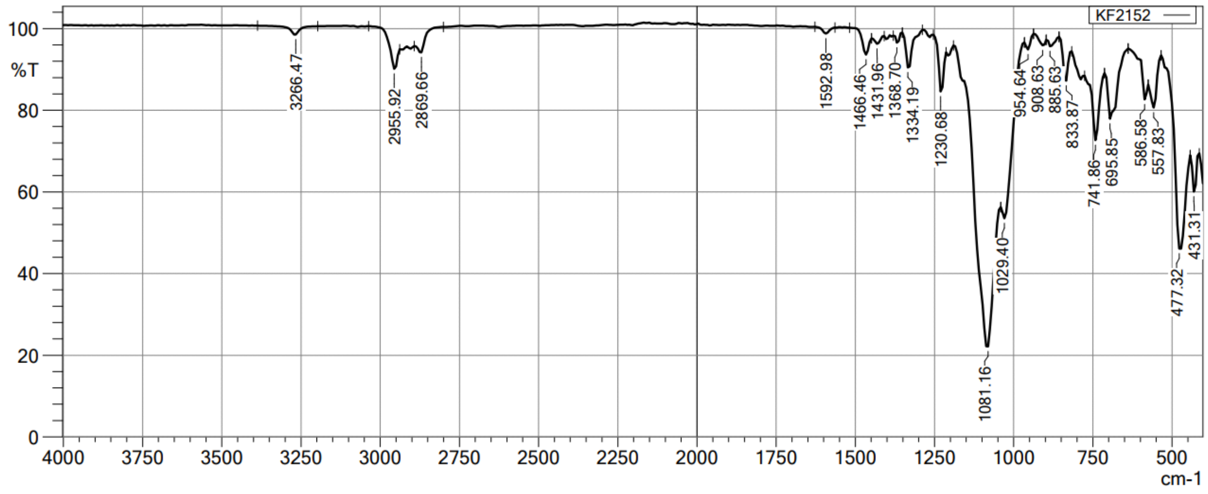

Figure S29. FT-IR spectrum of **5**.

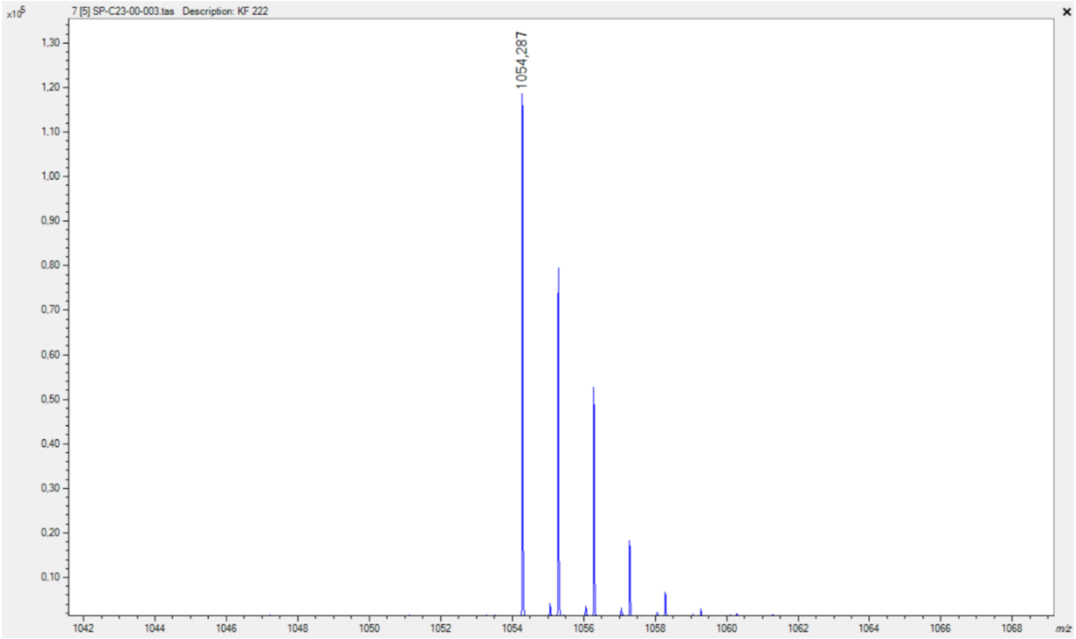

Figure S30. MALDI-MS ([M + Na]$^{+}$) of **5**.

	%C	%H	%N	%S
Calculated	43.03	7.22	1.36	3.10
**Measured**	**42.98**	**7.16**	**1.40**	**3.12**

Figure S31. Elemental analysis of **5**.

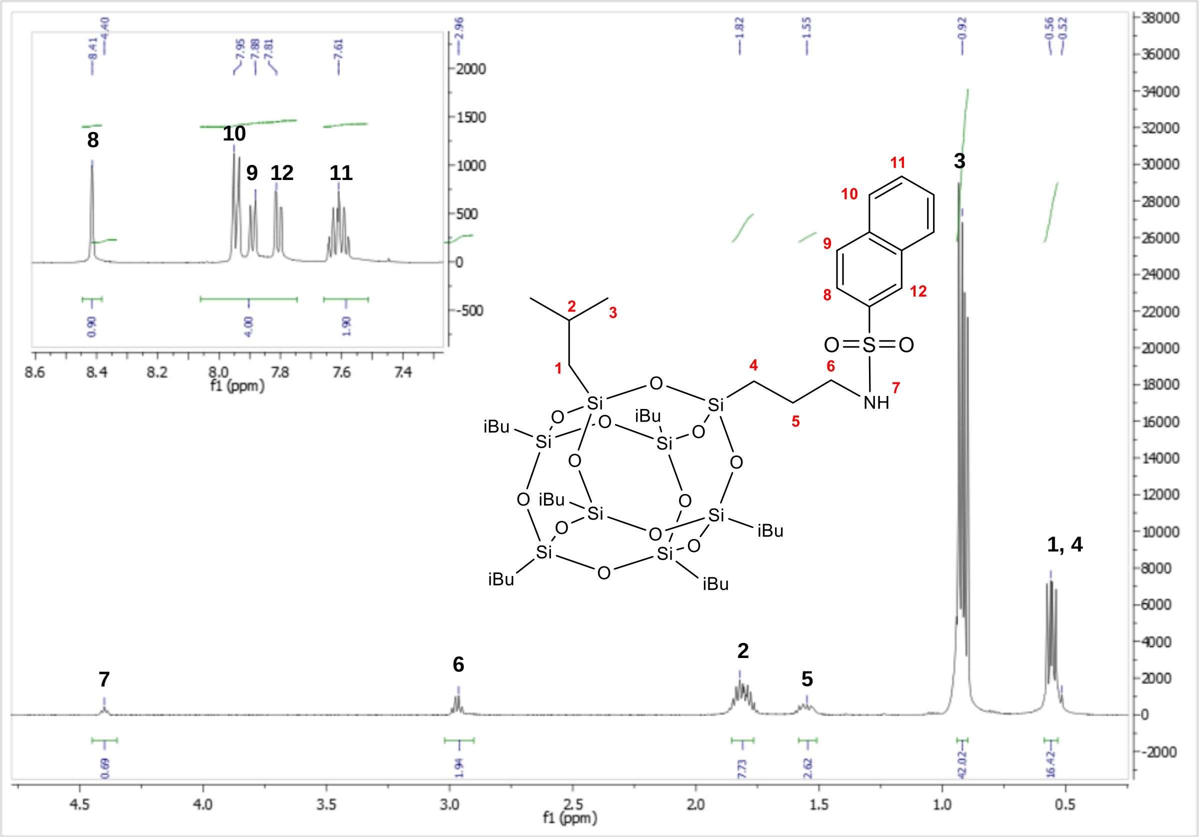

Figure S32. ^{1}H NMR (500 MHz, $CDCl_3$, 300 K) spectrum of **6**.

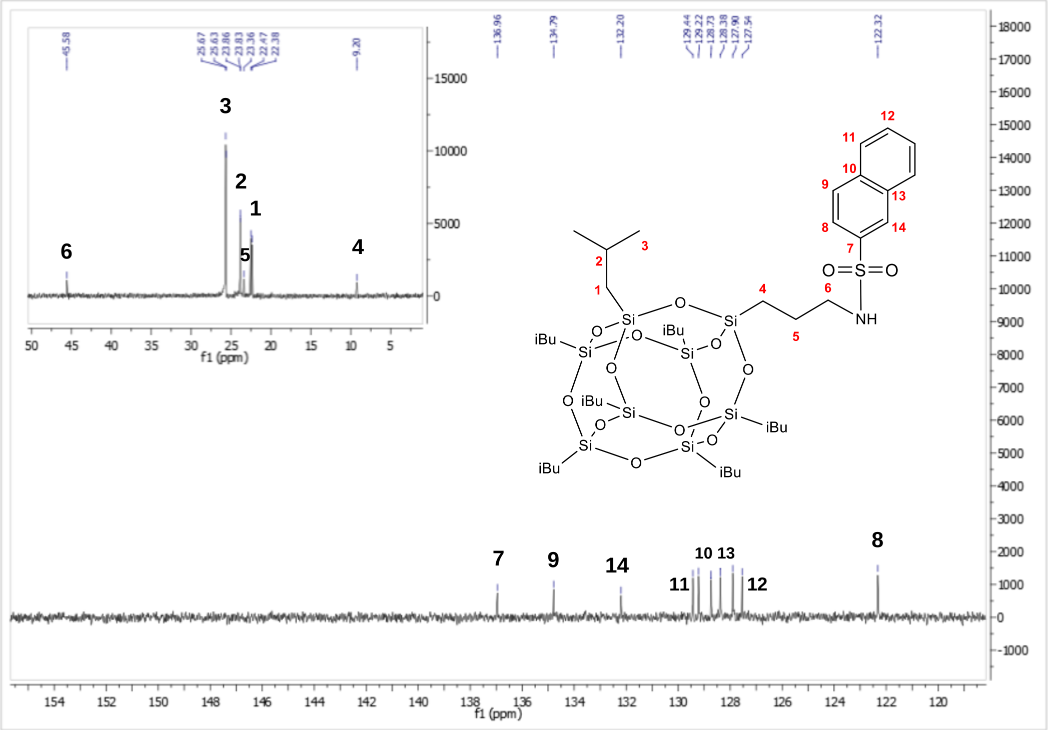

Figure S33. ^{13}C NMR (126 MHz, $CDCl_3$, 300 K) spectrum of **6**.

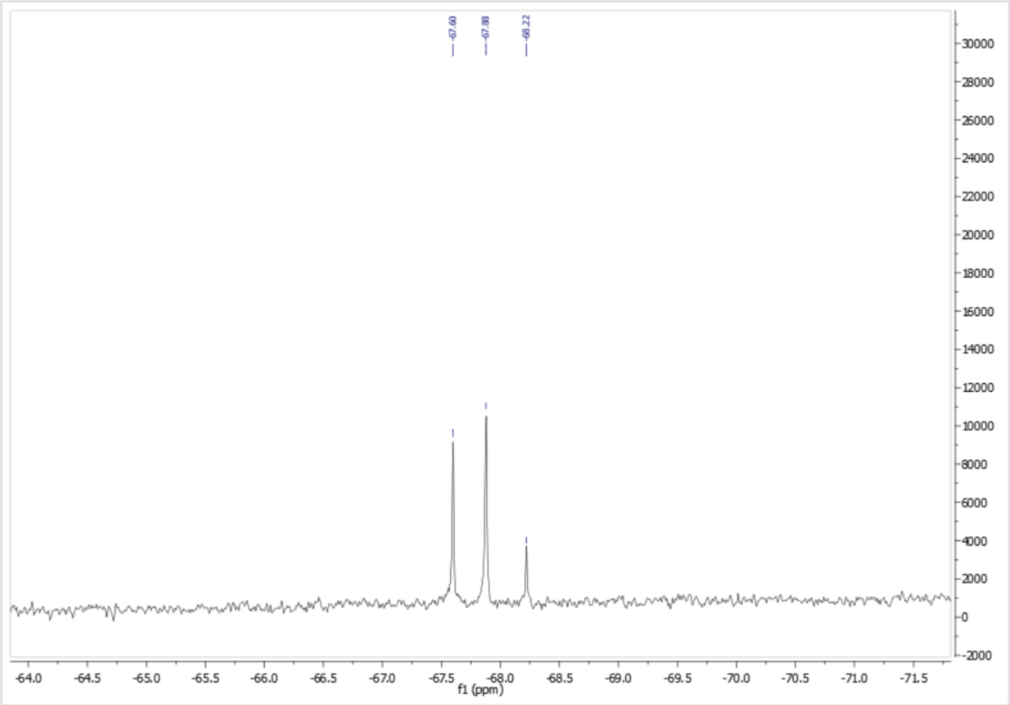

Figure S34. ^{29}Si NMR (99 MHz, $CDCl_3$, 300 K) spectrum of **6**.

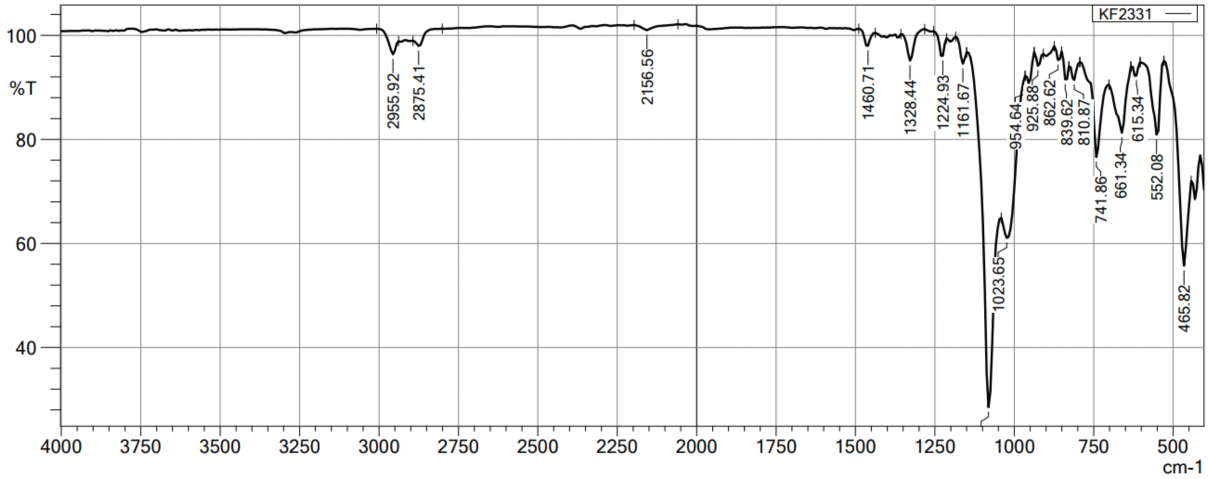

Figure S35. FT-IR spectrum of **6**.

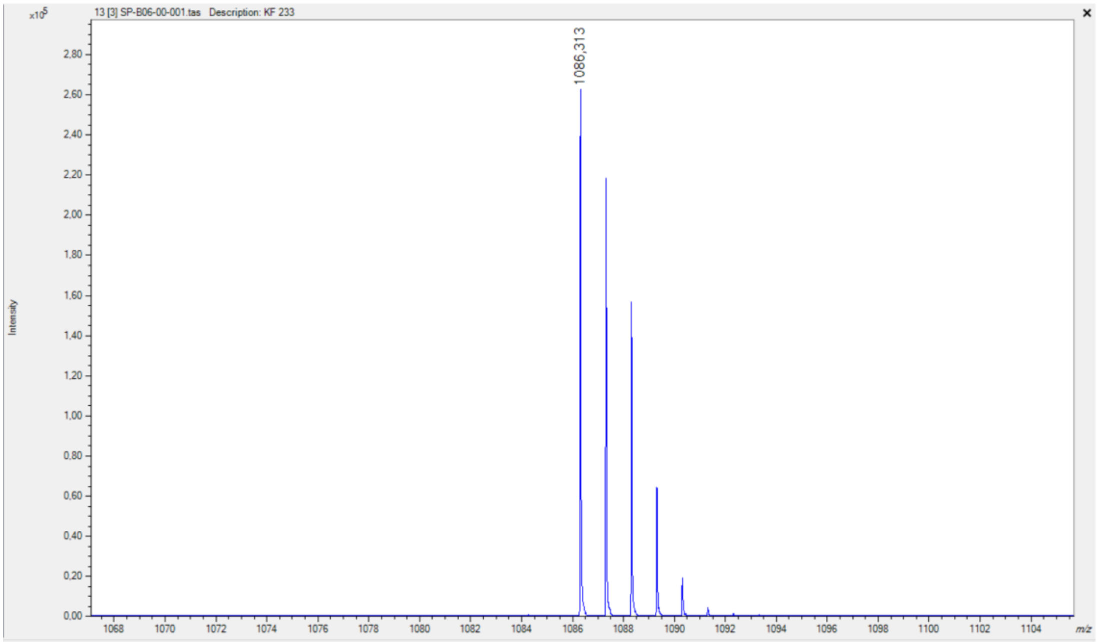

Figure S36. MALDI-MS ($[M + Na]^+$) of **6**.

	**%C**	**%H**	**%N**	**%S**
Calculated	46.25	7.29	1.32	3.01
**Measured**	**46.51**	**7.43**	**1.24**	**2.93**

Figure S37. Elemental analysis of **6**.

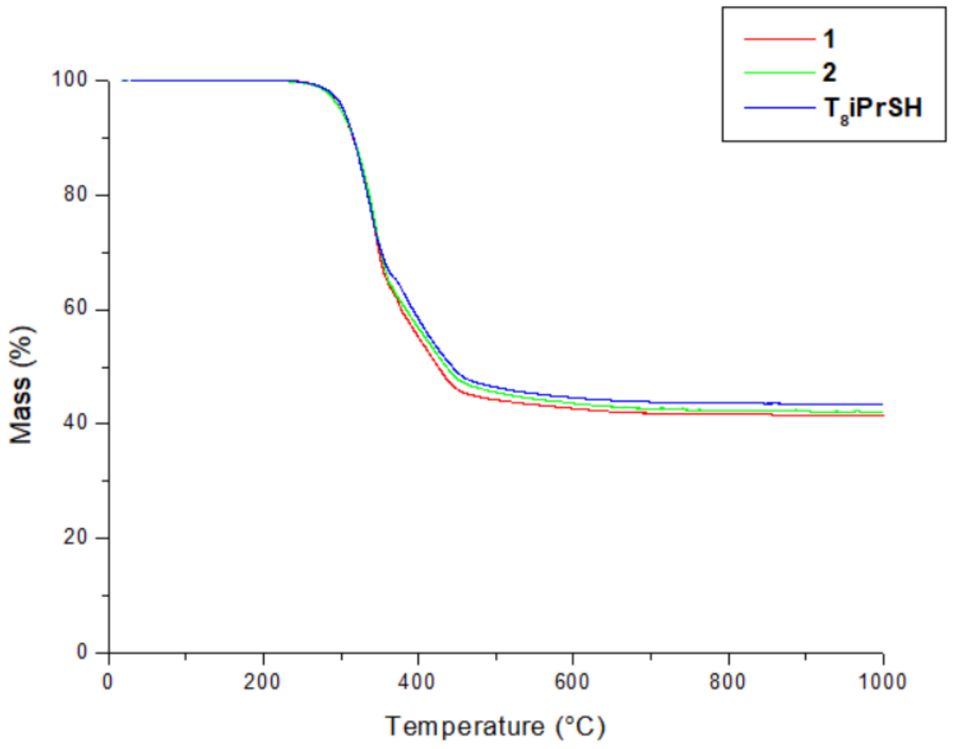

Figure S38. Thermal gravimetric analysis (TGA) profiles of **1**, **2** and their substrate – **T_8iPrSH**.

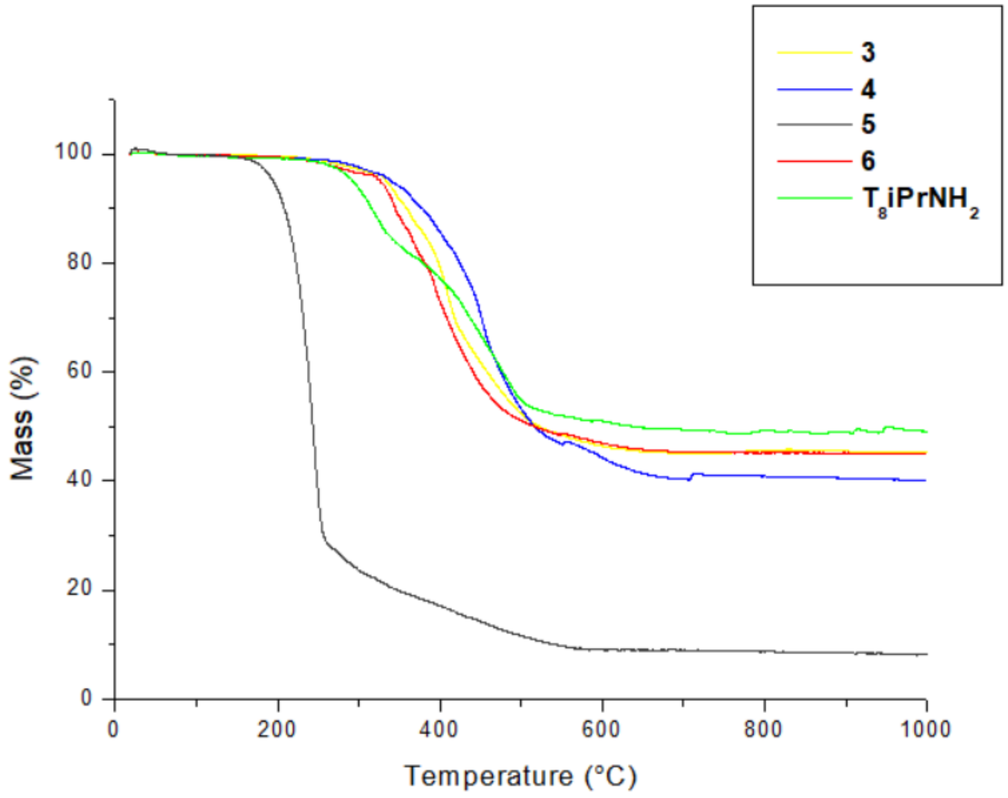

Figure S39. Thermal gravimetric analysis (TGA) profiles of **3**, **4**, **5**, **6** and their substrate – **T_8iPrNH$_2$**.

Table S2. The solubility of **1-6** in common solvents.

	**1**	**2**	**3**	**4**	**5**	**6**
**Diethyl ether**	Soluble	Soluble	Partially soluble – cloudy solution. No changes upon heating	Partially soluble – cloudy solution. No changes upon heating	Soluble	Partially soluble – cloudy solution. No changes upon heating
**Hexane**	Soluble	Soluble	Insoluble. A small amount dissolves upon heating	Insoluble. A small amount dissolves upon heating	Partially soluble – clear solution, some precipitate did not dissolve. No changes upon heating	Partially soluble – cloudy solution. No changes upon heating
**Ethanol**	Partially soluble – cloudy solution. No changes upon heating	Partially soluble – cloudy solution. No changes upon heating	Partially soluble – cloudy solution. No changes upon heating	Partially soluble – cloudy solution. No changes upon heating	Partially soluble – clear solution, some precipitate did not dissolve. No changes upon heating	Partially soluble – cloudy solution. No changes upon heating
**Water**	Insoluble	Insoluble	Insoluble	Insoluble	Insoluble	Insoluble
